# Hydrogel Enhanced Organoid Multidirectional Differentiation via Yap/Tead4 Mechanotransduction for Accelerated Tissue Regeneration

**DOI:** 10.1021/acsami.5c06161

**Published:** 2025-06-23

**Authors:** Peng Luo, Yuning Cheng, Yuwen Luo, Nan Zhang, Jingjing Cao, Honggang Wang, Xieyuan Jiang, Qian Wang, Xinbao Wu, Yajun Liu, Jianping Mao, Xinhua Zhou, Jing-Jun Nie, Dafu Chen

**Affiliations:** † Laboratory of Bone Tissue Engineering, Beijing Laboratory of Biomedical Materials, National Center for Orthopaedics, Beijing Research Institute of Traumatology and Orthopaedics, 66526 Beijing Jishuitan Hospital, Capital Medical University, Beijing 100035, China; ‡ Department of Orthopedic Trauma, National Center for Orthopaedics, Beijing Jishuitan Hospital, Capital Medical University, Beijing 100035, China; § Department of Spine Surgery, National Center for Orthopaedics, Beijing Jishuitan Hospital, Capital Medical University, Beijing 100035, China; ∥ Department of Orthopedic Surgery, National Center for Orthopaedics, Beijing Jishuitan Hospital, Capital Medical University, Beijing 100035, China

**Keywords:** Multiple organs repair, ECM mimic hydrogel, Organoid, Multidirectional differentiation, Yap/Tead4 mechanotransduction

## Abstract

The repair of multiple organs in motor systems remains a major clinical challenge that necessitates bioactive grafts with a multidirectional differentiation ability. Hydrogel-based organoids implants have emerged as pivotal tools and attracted great attentions. However, strategies to unlock the multipotency of bone marrow mesenchymal stem cells (BMSCs) by precisely modulating the mechanical and structural characteristics of biomimetic extracellular matrix (ECM) during hydrogel-based organoid construction remain underexplored. In this study, a gelatin methacryloyl (GelMA)-based biomimetic ECM mimic hydrogel (HG-2) loaded with BMSCs was developed to construct a multidirectional differentiation organoid, HG-2/3d-BMSC. The hydrogel could provide spatial mechanical stimulation to adherent BMSCs via cell adhesion induced cytoskeleton assembly. RNA sequencing (RNA-Seq) combined with in vitro and in vivo biological experiments reveals that ECM mimic hydrogels deliver adhesion-based spatial mechanical stimulation. This mechanical stimulation specifically unlocks the multipotency of BMSCs during osteogenic differentiation induction. Furthermore, it accelerates and enhances the multidirectional differentiation capacity of BMSCs, simultaneously promoting their commitment to osteogenic, chondrogenic, and tendonogenic tissue lineages. Further investigations prove that adhesion-based spatial mechanical stimulation from the ECM mimic hydrogel enhances multidirectional differentiation of BMSCs-based organoid via Yap/Tead4 (yes-associated protein/TEA domain transcription factor 4) mechanotransduction mediated Kat7 downregulation. The work not only advances the theoretical framework for designing biomaterials that exploit mechanical cues to override biochemical-driven lineage commitment but also establishes a novel paradigm for developing multifunctional organoid constructs to address the clinical challenge of regenerating hierarchically complex tissues in a motor system.

## Graphical abstract

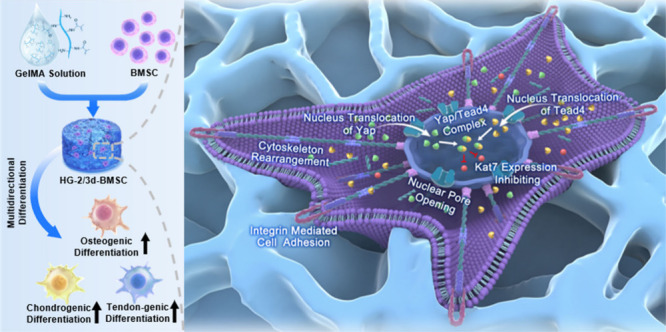

## 1 Introduction

Injuries caused by accidents, tumors, and aging can lead to severe trauma or dysfunction of multiple organs involved in the motor system, which cause serious socio-economic burdens.^1–5^ For example, acute knee joint injuries may encompass simultaneous tears of the anterior cruciate ligament, patellar tendon, and lateral meniscus;^4^ sprain-induced injuries can lead to ligamentous, tendinous, cartilaginous, and osteochondral damage in the ankle joint.^5^ Various inert or bioactive tissue implants have been developed for multiple organ injury therapy involved in the motor system. However, the repair of multiple organ injuries remains a major clinical challenge, necessitating an integrated surgical approach to ensure optimal clinical and functional recovery.

Multiple organ injuries of the motor system often involve extensive loss of tissues and structures. Organoids, as three-dimensional cell culture systems, rely on the interplay between stem cells and biomimetic three-dimensional (3D) extracellular matrices to simulate the cellular composition, spatial architecture, and physiological functions of native organs, emerging as pivotal tools in regenerative medicine.^6–8^ Notably, bone marrow mesenchymal stem cells (BMSCs)-based organoids have demonstrated remarkable efficacy in motor system regeneration.^9,10^ For instance, BMSCs-loaded microspheres subjected to chondrogenic induction in vitro can form callus organoids, enhancing chondrogenic efficiency.^9^ Conventional organoid construction strategies predominantly rely on biochemical stimulation, which efficiently drives BMSCs differentiation into specific lineages, and their capacity for multitissue repair in complex injuries remains to be explored.

The limited differentiation of organoids may arises from the over-reliance of traditional organoid construction methods on biochemical cues for directed differentiation.^11,12^ Conventional extracellular matrix (ECM) biomimetic strategies for organoid construction predominantly focus on mimicking the biochemical composition of natural tissues but often overlook the critical influence of mechanical properties and spatial structures on cellular behavior.^13–15^ Recent advancements in bioactive regenerative materials—including scaffolds, microgels, and hydrogels—have expanded the scope of biomimetic ECM engineering.^16,17^ Among these, hydrogels stand out as elastic 3D network materials with broadly tunable mechanical properties, adaptable spatial configurations, and exceptional biocompatibility, garnering significant attention.^18,19^ However, strategies to unlock the multipotency of BMSCs during organoid construction remain underexplored. These approaches require precise modulation of mechanical and structural characteristics in hydrogel-based biomimetic ECM, particularly for achieving multitissue regeneration in complex injuries.

Therefore, we propose a mechano-bioengineering strategy for constructing organoids with multitissue regeneration ability. ECM mimic hydrogel (HG-2) with optimized mechanical properties and spatial structures conducive to BMSCs survival and multipotency were prepared (Figure 1). Bioinformatics analysis and in vitro biological experiments confirmed that mechanical stimulation from HG-2 significantly enhanced and accelerated the multilineage differentiation capacity of encapsulated BMSCs. Subsequent in vivo experiments further demonstrated the remarkable ability of the HG-2/3d-BMSC organoid to synchronously regenerate bone, cartilage, and tendon tissues, showcasing promising potential for repairing compound motor system injuries. Moreover, we identified that Yap/Tead4 (yes-associated protein/TEA domain transcription factor 4)-mediated mechanotransduction coupled with Kat7 downregulation served as the pivotal regulatory axis through which ECM mimic hydrogel-derived spatial mechanical cues potentiated BMSCs multipotency. Our study not only elucidated the Yap/Tead4/Kat7 axis as a fundamental mechanobiological pathway governing stem cell fate determination but also established a novel paradigm for developing multifunctional organoid constructs to address the clinical challenge of regenerating hierarchically complex tissues in the motor system.

**Figure 1:**
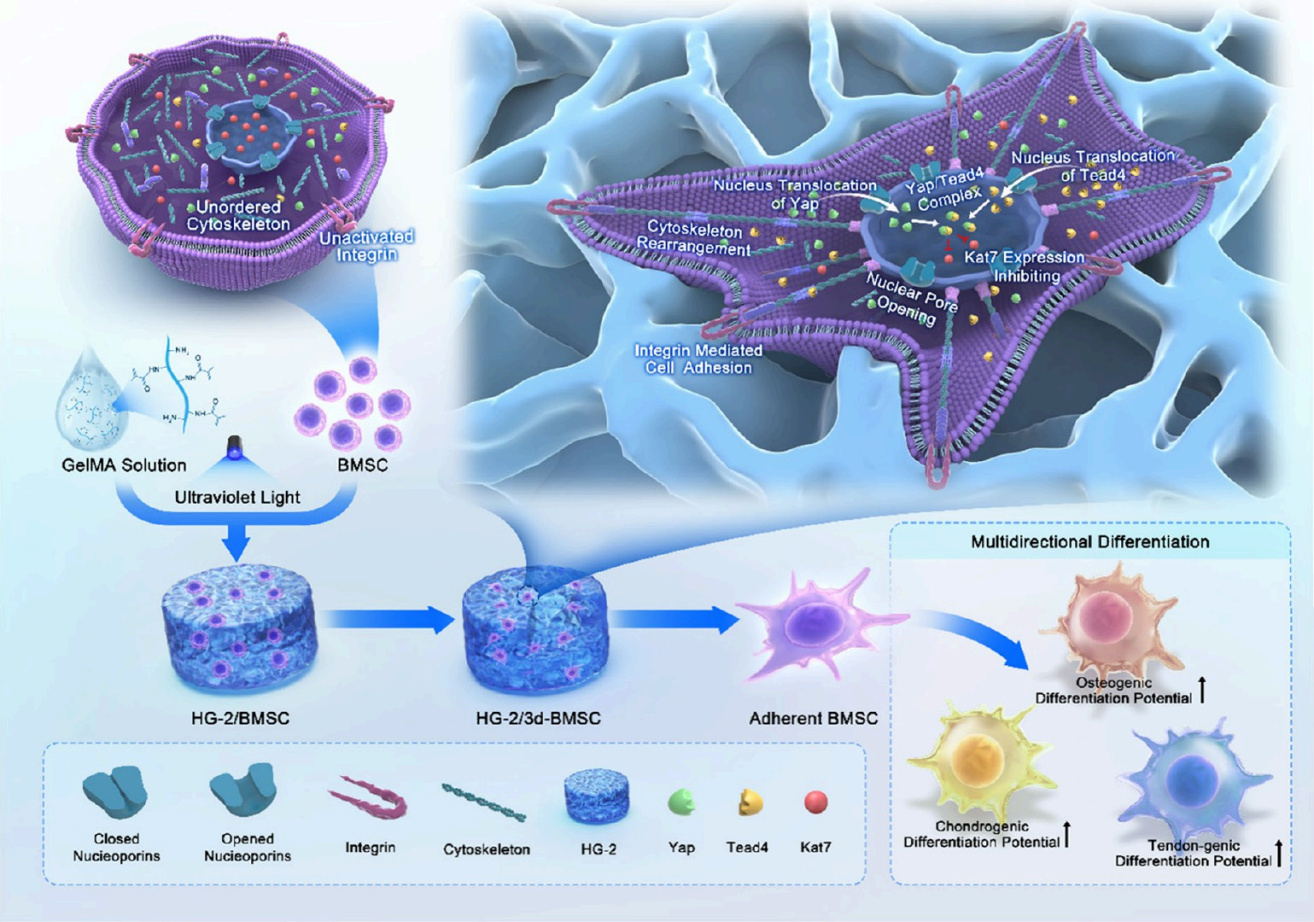
Schematic diagram showing the construction of organoid (HG-2/3d-BMSC) with multidirectional differentiation.

## 2 Experimental Section

### 2.1 Preparation of the Cross-Linked Gelatin Methacryloyl (GelMA)-Based ECM Mimic Hydrogel

The cross-linked GelMA-based ECM mimic hydrogel was prepared according to the GelMA kit protocols (Engineering for Life, China). Briefly, the initiator lithium phenyl-2,4,6-trimethylbenzoylphosphinate (LAP) was dissolved in a water bath at 40–50 °C with PBS to prepare 0.25% initiator standard solution. Hydrogels with the mass volume fraction of 7.5% (HG-1), 10% (HG-2), and 12.5% (HG-3) were prepared. For the preparation of HG-2 hydrogel, every 1 g of freeze-dried GelMA polymer was dissolved with 10 mL of the initiator standard solution in a water bath at 60–70 °C for 20–30 min in the dark. The obtained GelMA solution was then filtered with a 0.22 μm filter membrane prior to being added into a Teflon mold with a diameter of 10 mm and a depth of 2 mm. The mold with GelMA solution was irradiated with a 405 nm light source for 20 s to get the cross-linked GelMA-based ECM mimic hydrogel.

### 2.2 Characterization of the Cross-Linked GelMA-Based ECM Mimic Hydrogel

#### 2.2.1 Porous Morphology of Hydrogels

The GelMA-based ECM mimic hydrogel was freeze-dried and broken in liquid nitrogen. The porous morphology of hydrogels was observed using a scanning electron microscope (SEM) (Jeol, Japan). ImageJ software was used for the pore size of hydrogels measurement and analysis (*n* = 4).

#### 2.2.2 Swelling Property Evaluation

The swelling property of the GelMA-based ECM mimic hydrogel was evaluated by weighing. After freeze-drying in vacuum for 24 h, the dry weight (*W*_d_) of the sample was recorded and put into the well plate. The sample was immersed in 2 mL of sterile PBS at 37 °C, and excess PBS was removed after 1, 2, 4, 6, 12, and 24 h incubation. The sample was then weighed to obtain the swelling weight (*W*_s_). Swelling ratio = (*W*_s_ – *W*_d_)/*W*_d_ × 100% (*n* = 4).

#### 2.2.3 Degradation Assay of the Hydrogels

The degradation ability of GelMA-based ECM mimic hydrogel was tested in vitro under collagenase.^20,21^ The weight of GelMA hydrogel sample was weighed and recorded as *W*_0_, and then the sample was immersed in 2 mL of collagenase solution (2 U/mL) at 37 °C; all of the liquid was removed at 0, 1, 2, 4, 6, 12, and 24 h, and the excess liquid of the sample was removed by absorbent paper. The sample was then weighed to record the sample weight *W*_*n*_ (the sample weight at the serial time point was *W*_*n*_). Degradation ratio = (*W*_0_ – *W*_*n*_)/*W*_0_ × 100% (*n* = 4).

#### 2.2.4 Compression Resistance Evaluation

The GelMA-based ECM mimic hydrogel was placed on the platform of an electronic universal testing machine (Shimadzu, Japan) and subjected to compression tests at a loading rate of 0.2 mm/min until the sample rupture. The temperature was maintained at 25 °C during the test. The compression modulus was obtained by calculating the slope of the linear region of the stress–strain curve of the sample (*n* = 4).

#### 2.2.5 Rheological Characterizations

GelMA-based ECM mimic hydrogel was placed on a rheometer (Waters, America) test parallel plate to carry a small amplitude frequency scanning mode (temperature, 25 °C; strain amplitude, 0.1%; frequency range, 1–100 rad/s). The storage modulus (*G*′), loss modulus (*G*″), and viscosity (*n* = 4) were recorded.

#### 2.2.6 Hemocompatibility of Hydrogels

GelMA-based ECM mimic hydrogel was immersed in 2 mL of saline at 37 °C for 24 h to obtain extraction. A total of 950 μL of the extraction was mixed with 50 μL of rabbit erythrocytes (Solarbio, China) and incubated at 37 °C for 1 h. Triton X-100 was incubated with rabbit erythrocytes as the positive control, and normal saline was incubated with rabbit erythrocytes as the negative control. At the end of the incubation, the rabbit erythrocytes were centrifuged (3000 rpm, 5 min) and the absorbance of the supernatant was measured at 540 nm by Multiskan GO (Thermo Scientific, USA). Hemolysis ratio = (OD_specimen_ – OD_negative_)/(OD_positive_ – OD_negative_) × 100% (*n* = 4).

#### 2.2.7 Porosity Property Evaluation

The porosity property of the GelMA-based ECM mimic hydrogel was evaluated by measuring volume and mass. After freeze-drying in vacuum for 24 h, the dry weight (*W*_d_) of the sample was recorded and put into the well plate. The sample was immersed in 2 mL of sterile PBS at 37 °C, and excess PBS was removed after 24 h incubation. The sample was then weighed to obtain the swelling weight (*W*_s_). The sample was then measured in volume to obtain the swelling volume (*V*_s_). Swelling ratio = ((*W*_s_ – *W*_d_)/ρ_PBS_)/*V*_s_ × 100% (*n* = 4).

### 2.3 Cell Culture and Cell Viability

#### 2.3.1 Cell Culture

BMSCs used in this work were extracted from Sprague–Dawley rats and purchased from the Stem Cell Bank of Chinese Sciences Academy; The BMSCs were validated according to the International Society for Cell Therapy (ISCT) criteria for rat bone marrow mesenchymal stem cells by microscopic observation, surface marker profiling, and trilineage differentiation prior to delivery. The purchased BMSCs were cultured in a 37 °C incubator containing 5% CO_2_. The cells grew in a growth culture medium containing α-MEM medium (Gibco, USA), 10% fetal bovine serum (Gibco, USA), 1% penicillin–streptomycin (Gibco, USA), osteogenic differentiation induction medium containing α-MEM medium (Gibco, USA), 10% fetal bovine serum (Gibco, USA), 1% streptomycin mixture (Gibco, USA), β-glycerophosphoid sodium (Sigma, USA), dexamethasone (Sinopharm, China), and ascorbic acid (Sinopharm, China).

#### 2.3.2 Cell Viability Assay

The Live/Dead Cell Staining Kit (Solarbio, China) was used to assess the cell viability at different times. The stained HG-2 hydrogels with BMSCs culturing in growth culture medium on day 0 and day 14 were observed under an inverted fluorescence microscope. The live cells were marked in green fluorescence; dead cells were marked in red fluorescence.

### 2.4 Preparation of Different Organoids

The preparation procedures of different organoids are shown in Supporting Information Figure S3. Briefly, the BMSCs cultured in dishes were collected and divided into two parts. One part of the BMSCs was resuspended and added into a plate, followed by culturing in growth culture medium for 14 days. The cells were then collected and resuspended with HG-2 GelMA mixture at the density of 4 × 10^6^ cells/mL to get BMSCs loaded HG-2 hydrogels and cultured with osteogenic differentiation induction medium to obtain the type I organoid (termed as HG-2/BMSC group). The other part of the cells was directly resuspended with HG-2 GelMA mixture at the density of 4 × 10^6^ cells/mL, followed by culturing in growth culture medium for 14 days to get HG-2 hydrogels with fully attached and extended three-dimensional BMSCs, and then cultured with osteogenic differentiation induction medium to obtain the type II organoid (termed the HG-2/3d-BMSC group).

### 2.5 Adhesion of BMSCs in GelMA-Based ECM Mimic Hydrogel

Cytoskeleton/nucleus staining of the HG-2 hydrogels with BMSCs culturing in growth culture medium were performed to observe the morphology and adhesion of the cells in the ECM mimic hydrogel. The washing solution containing 0.1% TritonX-100 (Solarbio, China) was prepared with PBS, and bovine serum albumin (BSA) (Solarbio, China) was added to the above washing solution to prepare an antibody diluent containing 5% BSA and 0.1% TritonX-100. Actin-Tracker Red-555 (Beyotime, China) and antidilution solution were prepared at a ratio of 1:100 to prepare cytoskeleton staining work solution. After the sample was washed three times, DAPI staining solution (Beyotime, China) was added and the sample incubated in the dark for 5 min. The stained complexes were observed and photographed under an inverted fluorescence microscope. Red fluorescence marked the cytoskeleton, and blue fluorescence marked the nucleus.

### 2.6 Bulk RNA Sequencing (RNA-seq) of Different Groups

The HG-2/3d-BMSC group and the HG-2/BMSC group were both cultured with osteogenic differentiation induction medium for 3, 7, and 14 days. Then the hydrogel–cell complexes were lysed with GelMA lysate (Engineering for Life, China) and cells were obtained for the following assessments. Total RNA in the cells was extracted and tested for quality. The qualified RNA samples were transcribed to form a library, and the cDNA library was sequenced by an Illumina high-throughput sequencing platform. The raw sequenced reads with adaptor contamination more than 5 bp, quality value *Q* ≤ 19 accounting for more than 50% of the total bases, and N containing more than 5% in the raw data, were filtered. Corresponding analysis including gene expression level analysis (correlation analysis, principal component analysis) and differential gene enrichment analysis (GO enrichment analysis, KEGG enrichment analysis, transcriptome time sequence analysis, key pathway gene analysis) were performed on the mRNA-seq analysis cloud platform (Easyresearch Tech, China).

### 2.7 qRT-PCR Experiments of Different Groups

The extracted RNA was also reverse transcribed into cDNA by the reverse transcription kit (Vazyme, China), and the amplified cDNA and primers (Table S1) were assessed through qRT-PCR to detect the mRNA expression levels of differential genes including OSTERIX, OCN, ACAN, COL2A1, SCX, TNMD, TEAD4, CTGF, ANKRD1, and KAT7 by SYBR Mix (Vazyme, China) in Archimed X (Rocgene, China); the housekeeping gene was β-actin (*n* = 3).

### 2.8 Immunofluorescence Staining and Quantitative Analysis

The obtained grafts in different groups after being cultured with osteogenic differentiation induction medium for 3 days were washed with PBS and fixed with 4% (w/v) paraformaldehyde (Solarbio, China) for 20 min, and then it was permeated with 0.2% (w/v) Triton X-100 for 10 min at room temperature. They were then immersed in 5% (w/v) bovine serum albumin (BSA) for 1 h at room temperature. Primary antibodies were diluted in 5% (w/v) BSA, and more details about the antibodies were shown in Table S2. Samples were incubated with diluted primary antibody overnight at 4 °C, followed by incubation in goat antirabbit IgG Alexa Fluor 488 (Servicebio, 1:400) solution diluted in 5% (w/v) BSA for 1 h in the dark. Finally, the cells were incubated with DAPI staining solution (Beyotime, China) in the dark for 10 min and observed with fluorescence microscope. Red fluorescence marked the cytoskeleton or Tead4, green fluorescence marked the target protein, and blue fluorescence marked the nucleus. ImageJ software was used to detect the fluorescence intensity of the target protein for relative quantitative analysis; the fluorescence intensity of F-actin was used as the internal reference.

### 2.9 Animal Experiments

In this study, 6-week-old SPF Nude BALB/C immunodeficient mice (nude mice) were used for bilateral back cell-hydrogel complex implantation and approved by the ethics committee of Beijing Jishuitan Hospital (No. 202209-01). The hydrogel–cell complexes in the HG-2/3d-BMSC group and the HG-2/BMSC group were both cultured in osteogenic differentiation induction medium for 14 days prior to being implanted into the back of the mice. The samples in the HG-2 group were a GelMA-based ECM mimic hydrogel without BMSCs. A total of 12 nude mice in each group were cultured for 1, 2, 4, and 6 weeks, and 3 nude mice were sacrificed at each time point. The visceral and serum samples of nude mice at 6 weeks were collected to evaluate the biotoxicity of the implant materials in vivo.

### 2.10 Micro-CT Analysis

The implants removed from the back of immunodeficient mice after 4 and 6 weeks of incubation were examined using a high-resolution Mirco-CT scanning system (Skyscan, Belgium) to assess the mineralization ratio. Loop scanning was performed with the parameters set as voltage 49 kV and current 139 μA, and the slice thickness was 11.96 μm. The scanned data were imported into CTAn software (Bruker, Belgium) for three-dimensional reconstruction and analysis. The tissue volume, bone volume, and BV/TV of the cell–hydrogel complex were measured and analyzed.

### 2.11 Histological and Immunohistochemistry Staining

All samples taken from animals were sectioned at a thickness of 6 μm. Hematoxylin & eosin (H&E) staining was used to observe various cell types in the samples. Sirius red staining (under polarized light microscope) showed collagen fiber formation in the samples. Von Kossa staining was used to evaluate the deposition of calcium salt in the samples, and ImageJ software was used for quantitative analysis. In addition, immunohistochemical staining with anti-Osterix (Abcam, 1:1000) and anti-Ocn (Proteintech, 1:500) was used to detect the expression of bone regeneration-related proteins in the samples; immunohistochemical staining with anti-Acan (Proteintech, 1:500) and anti-Col2a1 (Proteintech, 1:1000) were used to detect the expression of cartilage regeneration-related proteins in the samples; immunohistochemical staining with anti-Scx (Thermo, 1:100) and anti-Tnmd (Abcam, 1:500) were used to detect the expression of tendon regeneration-related proteins in the samples; and ImageJ software was used for quantitative analysis.

### 2.12 Co-localization Analysis Based on Immunofluorescence Staining

The hydrogel-cell complexes in the HG-2/3d-BMSC group and the HG-2/BMSC group were cultured with osteogenic differentiation induction medium for 3 days. To further prove the function of GelMA-based ECM mimic hydrogel provided spatial mechanical stimulation by mediating BMSCs adhesion, 0.2 μΜ Cytochalasin D (MCE, USA) or 2 μΜ Verteporfin (MCE, USA) was added into the culture medium of the HG-2/3d-BMSC group. All of the obtained hydrogel–cell complexes were incubated with diluted primary antibodies shown in Table S2, followed by incubation in diluted goat antirabbit IgG Alexa Fluor 488 (Servicebio, 1:400) and goat antimouse IgG Cy3 (Servicebio, 1:400) solution. The DAPI staining was also applied to the stain nucleus. ImageJ software was used for fluorescence intensity localization analysis. The line tool was used to randomly select the target area and determine the fluorescence intensity of each point in the area, and the fluorescence intensity curve of each point in the area was drawn. The correlation between different fluorescence signals in the target region was quantified using Pearson’s correlation coefficient (PCC), which was calculated utilizing the Co-localization Finder plugin within ImageJ software. The resulting PCC values ranged from −1 to 1, where 1 indicated a perfect positive correlation, −1 represented a complete negative correlation, and 0 signified no correlation.

### 2.13 Cell Senescence Assay

The obtained grafts in different groups were obtained after culturing with osteogenic differentiation induction medium for 3 days. BMSCs seeded directly on the 24-well plate (plate group) or ECM mimic hydrogel surface (GelMA-coated group) were also prepared and cultured with osteogenic differentiation inducted for 3 days. Cell senescence β-galactosidase staining kit (Beyotime, China) was used to observe the cell senescence in different groups. Senescent cells are marked in blue. Total cell number and senescent cell number were counted using ImageJ software, and cell senescence ratio = number of senescent cells/total cell number × 100%.

### 2.14 Statistical Analysis

The experimental results were repeated 3 times or more and expressed as mean ± standard deviation (SD). Independent sample *t* test and one-way analysis of variance were used to analyze the differences between groups according to the needs of the situation. When *p* < 0.05, the difference was considered statistically significant, and the specific symbols were expressed as follows: ns means no significant difference; * for 0.01 < *p* < 0.05; ** for 0.001 < *p* < 0.01; *** for 0.0001 < *p* < 0.001; **** for *p* < 0.0001. Prism 9.5 software was used for data analysis and chart making.

## 3 Results and Discussion

### 3.1 Construction and Characterization of GelMA-Based ECM Mimic Hydrogel

GelMA is a biomaterial derived from gelatin, and has achieved extensive application in the construction of ECM mimic hydrogels.^21–24^ First, its hydrophilic gelatin-based network provides biocompatible microenvironments that effectively adsorb regenerative factors such as VEGF and FGF from the tissue niche, enabling sustained biochemical signal release to guide cell differentiation and tissue remodeling.^22^ Second, the RGD peptide motifs on GelMA surfaces specifically bind to integrin receptors on cell membranes, activating mechanotransduction pathways including FAK/PI3K signaling cascades, which enhance mechanical signal transmission efficiency through downstream Yap/Tead4 nuclear translocation.^23^ Crucially, GelMA establishes a multimodal synergy framework where its dual capacity for biochemical factor retention and integrin-mediated mechanical signaling converges to amplify cellular responses.^24^ Thus, GelMA was an excellent organoid scaffold candidate material. In this study, we fabricated GelMA-based hydrogels with three different mass volume fractions (w/v): 7.5% (HG-1), 10.0% (HG-2), and 12.5% (HG-3) to filtrate GelMA-based ECM mimic hydrogel (Figure S1A).

Different hydrogels exhibited characteristic porous structures under SEM observation, with pore sizes decreasing as the mass volume fraction increased. Specifically, the pore sizes reduced from 556.7 ± 157.5 to 173.6 ± 15.6 μm (Figure 2A and Figure S1B), and the porosity was also decreased from 89.98 ± 2.02 to 80.22 ± 1.51% (Figure S1C), which provided an optimal porous architecture that supports cell viability and facilitates substance exchange.^25,26^ Swelling capacity was important to the transport of nutrients and waste,^27^ and among the three hydrogels, HG-3 exhibited the lowest equilibrium swelling ratio while still maintaining adequate swelling capacity (Figure 2B).

**Figure 2:**
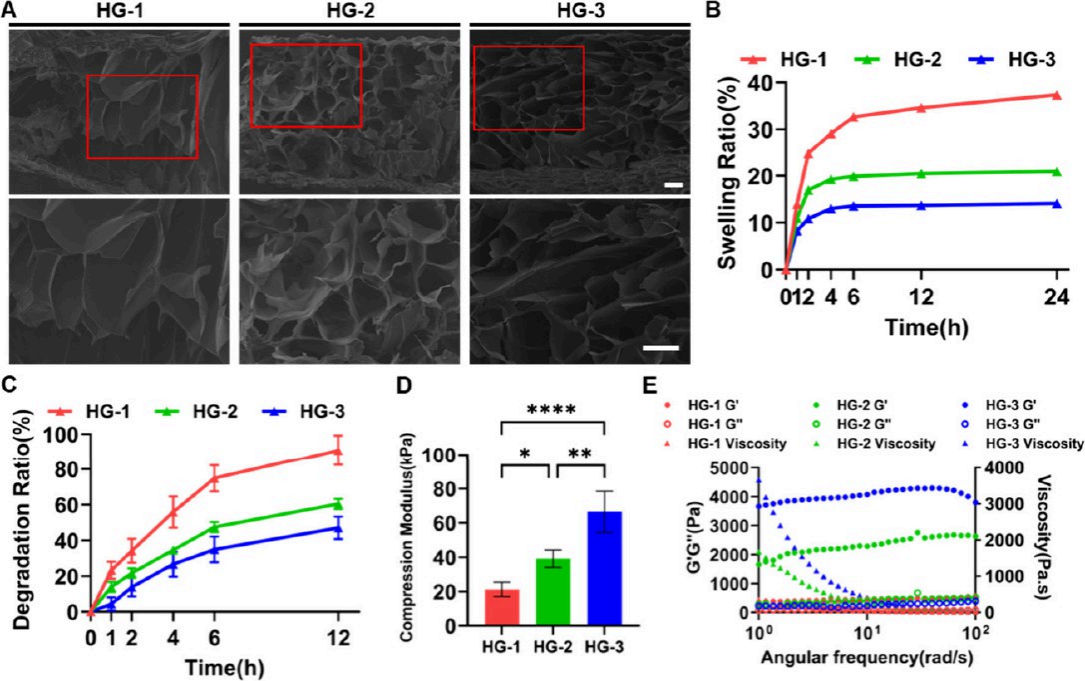
Biophysical characterization of the cross-linked GelMA-based ECM mimic hydrogel. (A) SEM images showing the porous ultrastructure of different hydrogels. Scale bar = 200 μm. (B, C) Swelling ratio and degradation ratio of different hydrogels. (D) Compression modulus showing the mechanical properties of different hydrogels, one-way analysis of variance, one-tailed. (E) Storage modulus (*G*′), loss modulus (*G*″), and viscosity showing the rheological properties of different hydrogels. All data are presented as mean ± SD (*n* = 4; *p < 0.05; **p < 0.01; ****p < 0.0001; ns means no significance).

The degradation ratios of HG-1, HG-2, and HG-3 hydrogels after 12 h were 90.8 ± 8.4, 60.5 ± 2.9, and 46.9 ± 6.3%, respectively (Figure 2C). This controlled degradation profile aligned with the requirements for tissue regeneration, providing adequate space for cytoskeleton extension and cell adhesion.^28^ We further observed that the compression modulus of various hydrogels increased significantly with higher GelMA mass volume fractions (Figure 2D); the modulus ranged from 21.2 ± 4.2 to 66.5 ± 12.1 kPa, providing an optimal matrix stiffness to promote stem cell differentiation.^29–31^

As has been reported, the stiffness of matrix between 25 and 40 kPa benefited the osteogenesis-related genes (such as RUNX2 and OSTERIX) activation of loaded human mesenchymal stem cells (hMSCs) and promoted their osteogenic differentiation,^29^ while 15–30 kPa stiffness matrix of the environment can induce cartilage cell differentiation;^30^ and 30–50 kPa stiffness can promote the expression of TENOMODULIN, SCX, which accelerated tendon differentiation.^31^ The stiffness of the prepared HG-1/-2/-3 hydrogels ranged from 21.2 ± 4.2 to 66.5 ± 12.1 kPa, which completely covered the optimal stiffness interval of the three differentiation directions mentioned above. In particular, the stiffness of HG-2 hydrogel was 39.1 ± 5.1 kPa, which was well within the optimal overlap between osteogenic (25–40 kPa) and tenogenic (30–50 kPa) differentiation and close to the upper limit of chondrogenic differentiation. Therefore, the HG-2 hydrogel provided a suitable mechanical microenvironment for multidifferentiation of stem cells into bone, cartilage, and tendon.

Moreover, low-viscosity hydrogels result in poor cell sedimentation and uneven cell distribution, while high-viscosity hydrogels ensure good cell uniformity by increasing the buoyancy acting on the cells.^32,33^ With the increase in GelMA mass volume fraction, the viscoelasticity of different hydrogels increased significantly, and the viscoelasticity of HG-2 was most conducive for the construction of the ECM mimic hydrogel (Figure 2E). The successful clinical application of this material relies on its satisfactory blood compatibility, which is considered as one of the crucial criteria.^34^ The hemolysis test showed that the hydrogels did not cause significant hemolysis (Figure S1D). Taken the above results together, we confirmed that HG-2 with 10.0% mass and volume fractions of GelMA exhibited suitable biophysical property and biocompatibility; thus, the HG-2 was chosen to construct ECM mimic hydrogel.

### 3.2 Adhesion and Proliferation of BMSCs in GelMA-Based ECM Mimic Hydrogel

We explored the time-dependent adhesion and survival of BMSCs in ECM mimic hydrogel at initial BMSCs density of 4 × 10^6^ cells/cm^3^ culturing in growth culture medium. As shown in Figure S2A, the cytoskeleton area of individual cells gradually increased with the culture time, and the cytoskeleton between cells was fully contacted at day 14, in other words, the BMSCs were fully extended and attached at this time in ECM mimic hydrogel. In addition, the results of live/dead cell staining in Figure S2B and corresponding live cell quantification in Figure S2C showed that the cells in the ECM mimic hydrogel survived well during 14 days in growth culture medium, but there was no significant increase in cell number. Therefore, we proposed that the cells loaded in ECM mimic hydrogel underwent extension and adhesion first rather than proliferation, and the process needed about 14 days.

The remarkable adhesion of BMSCs to ECM within the ECM mimic hydrogel provided a unique opportunity. This specific interaction not only enabled the physical attachment of BMSCs, but also permitted the meticulous construction of hydrogel–cell complexes, which potentiated the survival and multipotency of BMSCs. Such a specific interaction, carefully assembled in the context of an ECM mimic hydrogel, was furnished with the necessary biochemical and biomechanical cues essential for regulating the multidirectional differentiation of BMSCs in a controlled manner. It was through this adhesion process of BMSCs that the ECM mimic hydrogel served as a crucial scaffold to support the survival and functional activities of BMSCs.

### 3.3 Spatial Mechanical Stimulation from ECM Mimic Hydrogel Accelerates the Multidirectional Differentiation Process of Stem Cells

The reorganization of cytoskeleton in stem cells from short, disordered filaments into long, ordered structures could deliver spatial mechanical stimulation from ECM to the nuclear membrane.^35–37^ Thus, we proposed that fully extended BMSCs in the HG-2/3d-BMSC hydrogel–cell complex could sense and transmit spatial mechanical stimulation from the ECM mimic hydrogel through adhesion-mediated cytoskeleton rearrangement, which in turn regulated the BMSCs differentiation. To investigate the effect of spatial mechanical stimulation on the differentiation potential of BMSCs, we prepared two groups of hydrogel–cell complexes prior to osteogenic differentiation induction (Figure S3). RNA was extracted for transcriptome sequencing on days 3, 7, and 14 of osteogenic differentiation induction. First, we performed correlation analysis (Figure S4A) and principal component analysis (Figure S4B) on the sequencing results of two groups of RNA samples. The results showed that there were differences in the transcriptome of RNA samples from the HG-2/3d-BMSC group and the HG-2/BMSC group, and the transcriptome of samples in each group was highly similar, with good biological repeatability and high credibility.

The differential expressed genes (DEGs) between the HG-2/3d-BMSC group and the HG-2/BMSC group at different times of osteogenic differentiation induction was performed, and the experimental results were shown in Figure S5A–C. KEGG enrichment analysis and GO-BP enrichment analysis were further performed on the upregulated DEGs. In KEGG enrichment analysis, the upregulated DEGs between the HG-2/3d-BMSC group vs HG-2/BMSC group at each time point of osteogenic differentiation were mainly enriched in the pathways of extracellular matrix receptor interaction, protein digestion and absorption, and cell cycle (Figure S6). In the GO-BP enrichment analysis, the upregulated DEGs between HG-2/3d-BMSC group vs HG-2/BMSC group were mainly enriched in regeneration and repair of bone, cartilage, and tendon (Figure S7).

The changes of bone regeneration, cartilage regeneration, and tendon regeneration related to upregulated DEGs with time were analyzed and shown in a time trend transcriptome heat map (Figure S8) and a key gene heat map (Figure 3A). We found that the bone regeneration-related activity of BMSCs in the HG-2/3d-BMSC group was more active than that in the HG-2/BMSC group, and the difference increased with induction time. On the third day of induction, the cartilage regeneration-related activity of BMSCs in the HG-2/3d-BMSC group was less active than that in the HG-2/BMSC group, but the cartilage regeneration-related activity of BMSCs in the HG-2/BMSC group decreased gradually with induction time, while the cartilage regeneration-related activity of BMSCs in the HG-2/3d-BMSC group remained at a high level and was more active than that in the HG-2/BMSC group. The tendon regeneration-related activity of BMSCs in the HG-2/3d-BMSC group was more active than that in the HG-2/BMSC group, but the difference decreased with the induction time.

**Figure 3:**
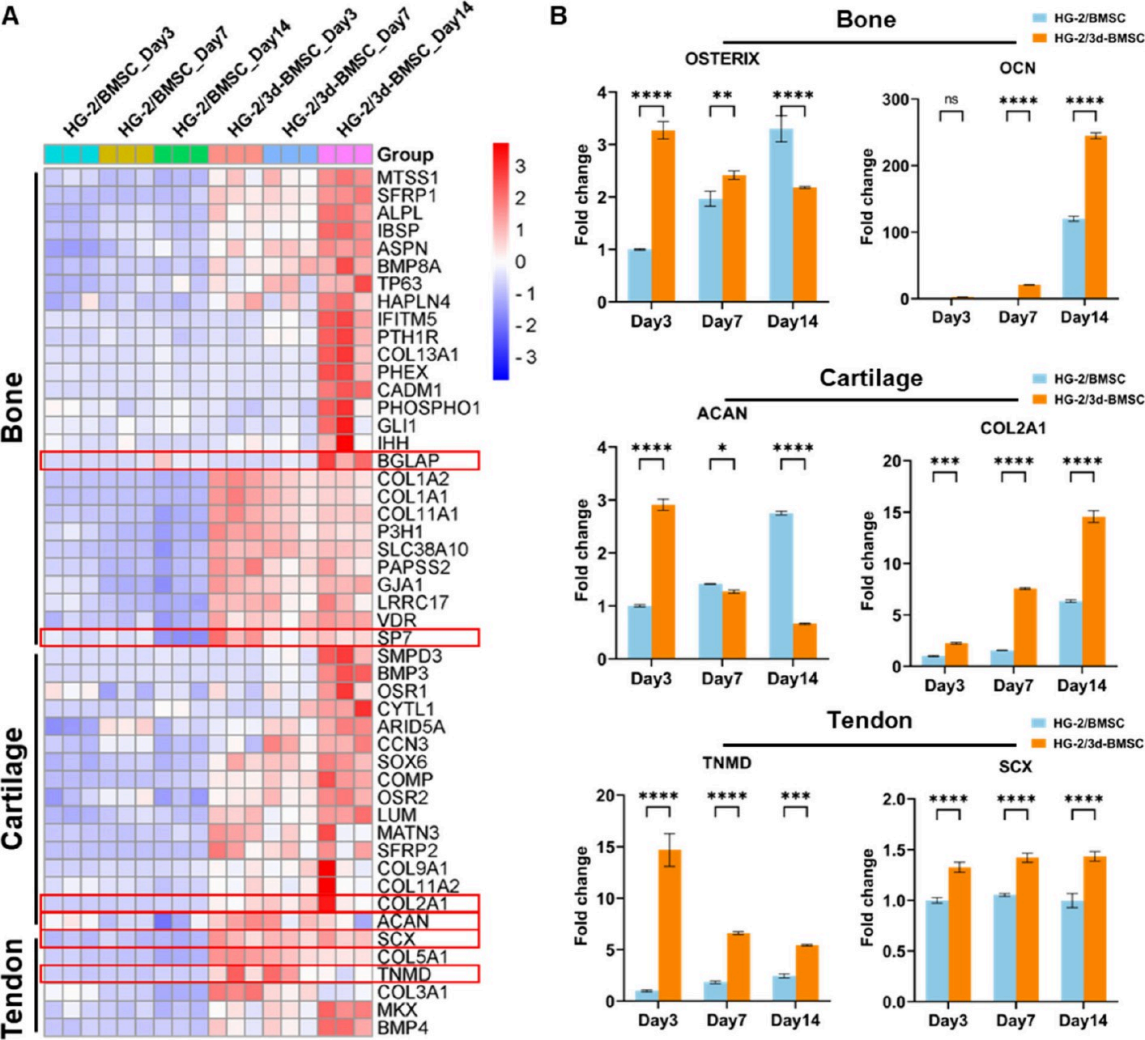
RNA-seq of BMSCs in different groups after osteogenic differentiation induction for different times. (A) Bone, cartilage, and tendon regeneration-related gene expressions of BMSCs after osteogenic differentiation induction for 3, 7, and 14 days, with red boxes indicating representative genes. (B) qRT-PCR analysis confirming the expression of OTSERIX, OCN, ACAN, COL2A1, TNMD, and SCX of BMSCs, independent sample *t* test, one-tailed. All data are presented as mean ± SD (*n* = 3; *p < 0.05; **p < 0.01; ***p < 0.001; ****p < 0.0001). HG-2/BMSC group, ECM mimic hydrogel with nonadherent BMSCs; HG-2/3d-BMSC group, ECM mimic hydrogel with adherent BMSCs.

The above results indicated that the BMSCs in the HG-2/3d-BMSC group showed enhanced and accelerated multidirectional differentiation ability. The typical activities of BMSCs were adhesion and cytoskeleton rearrangement rather than proliferation as soon as being loaded into the ECM mimic hydrogel, and the process needed about 14 days (HG-2/3d-BMSC) (Figure S2A–C). Then the spatial mechanical stimulation from ECM mimic hydrogel could be sensed by integrin-mediated cell adhesion and transmitted by the assembled cytoskeleton,^35,38,39^ and the well-transmitted spatial mechanical stimulation led to the accelerated transcription of genes associated with bone, cartilage, and tendon regeneration of BMSCs (Figure 3A and Figure S8).^40–42^

To confirm the sequencing results, representative genes of different differentiation phenotypes were tested by qRT-PCR (Figure 3B). The experimental results showed that the expressions of OSTERIX, ACAN, and TNMD in the HG-2/3d-BMSC group, which are expressed in the early stage of bone, cartilage, and tendon regeneration-related activities,^43–45^ were significantly higher than those in the HG-2/BMSC group. The results implied the repriming of BMSCs’ multidirectional differentiation in the HG-2/3d-BMSC group. The expression levels of early expressed genes decreased in the HG-2/3d-BMSC group and increased in the HG-2/BMSC group with induction time. The expression levels of OCN, COL2A1, and SCX in the HG-2/3d-BMSC group were always higher than those in the HG-2/BMSC group during the induction, and the expression levels of late expressed genes of bone, cartilage, and tendon regeneration-related activities of BMSCs in each group gradually increased with induction time.

Immunofluorescence staining and quantitative analysis of the proteins encoded by the above genes after 3 days of differentiation induction in the two groups were performed. The results showed that the positive ratio of fluorescence signals related to bone, cartilage, and tendon regeneration-related proteins in the HG-2/3d-BMSC group was higher than that in the HG-2/BMSC group (Figure 4A–D and Figures S9–S11). These findings proved that spatial mechanical stimulation from ECM mimic hydrogel could unlock the multipotency of BMSCs suppressed by osteogenic differentiation induction, thereby accelerating and enhancing the multidirectional differentiation process of BMSCs.

**Figure 4:**
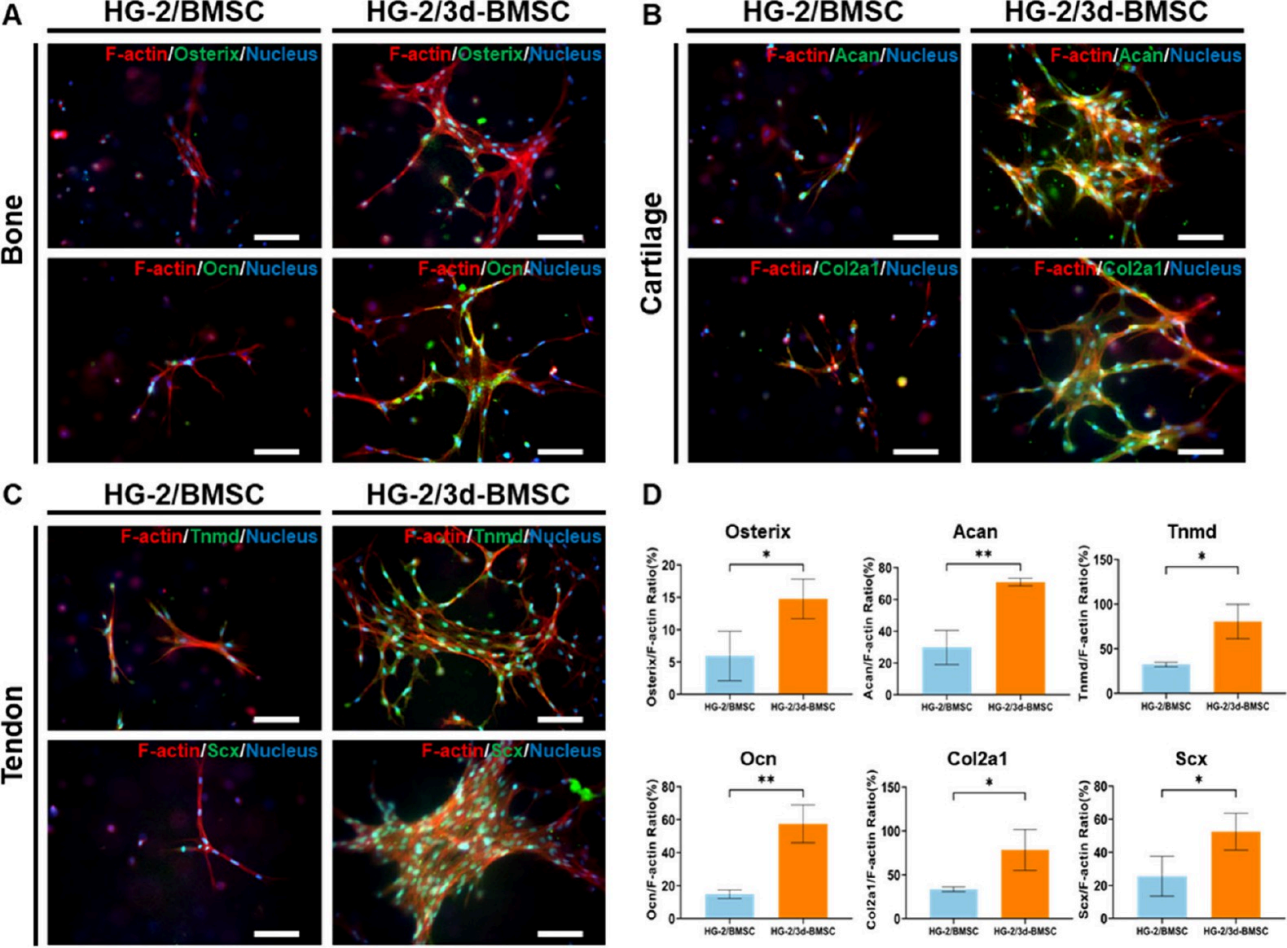
Differentiation ability of obtained grafts after osteogenic differentiation induction. Scale bar = 100 μm. (A) Immunofluorescence images of typical bone regeneration-related proteins (Osterix, Ocn) of BMSCs in different groups at day 3 after induction. (B) Immunofluorescence images of typical cartilage regeneration-related proteins (Acan, Col2a1) of BMSCs in different groups at day 3 after induction. (C) Immunofluorescence images of tendon regeneration-related proteins (Tnmd, Scx) of BMSCs in different groups at day 3 after induction. (D) Semiquantitative analysis of typical bone regeneration-related proteins (Osterix, Ocn), cartilage regeneration-related proteins (Acan, Col2a1), and tendon regeneration-related proteins (Tnmd, Scx) of BMSCs in different groups at day 3 after induction, independent sample *t* test, one-tailed. All data are presented as mean ± SD (*n* = 3; *p < 0.05; **p < 0.01). HG-2/BMSC group, ECM mimic hydrogel with nonadherent BMSCs; HG-2/3d-BMSC group, ECM mimic hydrogel with adherent BMSCs.

### 3.4 Spatial Mechanical Stimulation from ECM Mimic Hydrogel Promoting Multidirectional Differentiation Ability of Loaded BMSCs In Vivo

The hydrogel–cell complexes in the HG-2/3d-BMSC group and the HG-2/BMSC group were culture in osteogenic differentiation induction medium for 14 days. The obtained complexes were implanted subcutaneously into the backs of nude mice, and ECM mimic hydrogel without BMSCs were implanted subcutaneously into the backs of nude mice as a blank control (HG-2 group). We first stained the samples with H&E, which revealed the presence of various cell types in different groups. This result indicated the enhanced BMSCs differentiation in HG-2/3d-BMSC than the HG-2/BMSC group (Figure 5A and Figures S12 and S13A).

**Figure 5:**
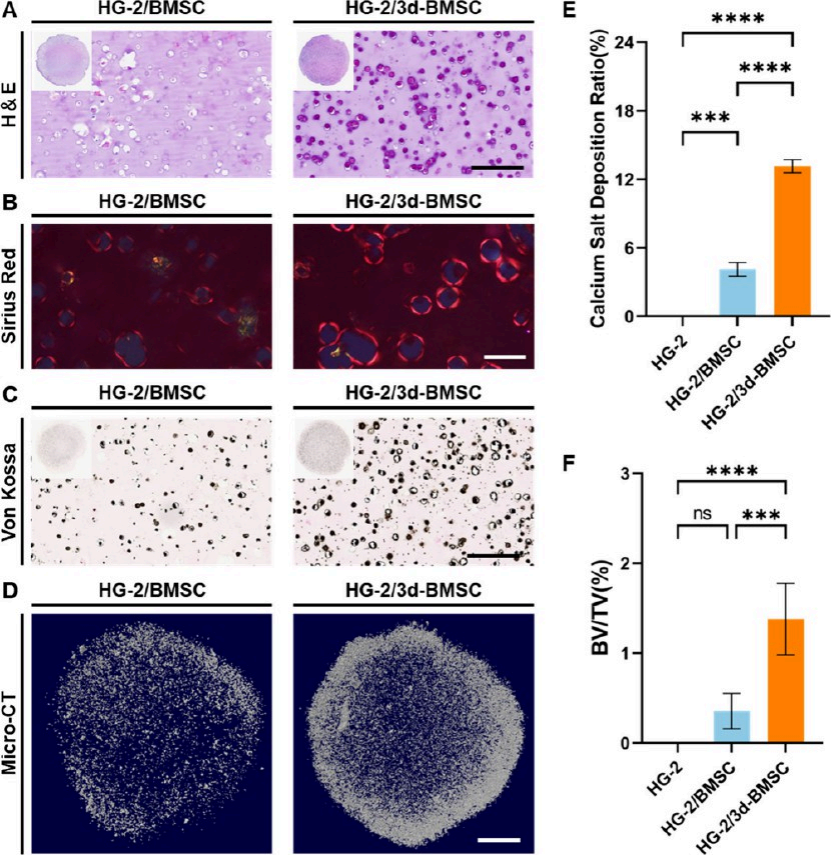
In vivo multidirectional differentiation ability of obtained grafts after osteogenic differentiation induction for 14 days. (A) H&E staining showing new tissue generation of different groups after 6 weeks. Scale bar = 200 μm. (B) Sirius red staining (under polarized light microscope) showing collagen fiber formation of different groups after 6 weeks. Scale bar = 50 μm. (C, E) Von Kossa staining and quantitative analysis results showing calcium salt deposition of different groups after 6 weeks, one-way analysis of variance, one-tailed. Scale bar = 200 μm. (D, F) Micro-CT scan reconstruction and quantitative analysis results providing the mineralization degrees of different groups after 6 weeks, one-way analysis of variance, one-tailed. Scale bar = 200 mm. All data are expressed as mean ± SD (*n* = 3; ***p < 0.001; ****p < 0.0001; ns means no significance). HG-2 group, ECM mimic hydrogel without BMSCs; HG-2/BMSC group, ECM mimic hydrogel with nonadherent BMSCs; HG-2/3d-BMSC group, ECM mimic hydrogel with adherent BMSCs.

The Sirius red staining results observed under a polarized light microscope showed that the HG-2/3d-BMSC group exhibited enhanced and accelerated collagen fiber formations compared to the HG-2/BMSC group (Figure 5B and Figures S13B and S14). We also observed the same change patterns of calcium salt deposition by using Von Kossa staining (Figure 5C,E and Figures S13C and S15). Reconstruction of Micro-CT results and quantitative analysis proved that the HG-2/3d-BMSC group possessed higher mineralization ability than the HG-2/BMSC group, and the mineralization ratio gradually increased with time (Figure 5D,F and Figures S13D and S16). Notably, the mineralization characteristics of the mineralized samples were more obvious in the peripheral part of the implant than in the central part of the implant, which was closely related to the physiological microenvironment of the subcutaneous tissue. The regions surrounding the implant are in more direct contact with the host vasculature than the central regions, creating more efficient gradients for nutrient transport (e.g., glucose, amino acids) and material exchange (e.g., oxygen).^46^ This difference in the microenvironment leads to more adequate nutrition and oxygen for cells in peripheral regions of the implant, which is conducive to the life activities of cells and promotes the mineralization of peripheral regions. In addition, higher interstitial fluid flow rates in the peripheral regions may reduce metabolic waste accumulation, maintain cell viability, and further accelerate mineralization deposition.^47^ Moreover, no obvious cells, collagen fibers, calcium salt deposition, or mineralization were observed in the HG-2 group.

IHC staining and corresponding quantitative analysis showed that the early bone, cartilage, and tendon tissue regeneration indicators including Osterix, Acan, and Tnmd in the HG-2/3d-BMSC group were lower than those in the HG-2/BMSC group, while the late indicators including Ocn, Col2a1, and Scx proteins were significantly higher in the HG-2/3d-BMSC group (Figure 6A–C and Figures S17–S23). The results confirmed the enhanced and accelerated multidirectional differentiation of BMSCs in the HG-2/3d-BMSC group than in the HG-2/BMSC group, which promoted the construction of organoid for multitissue regeneration.

**Figure 6:**
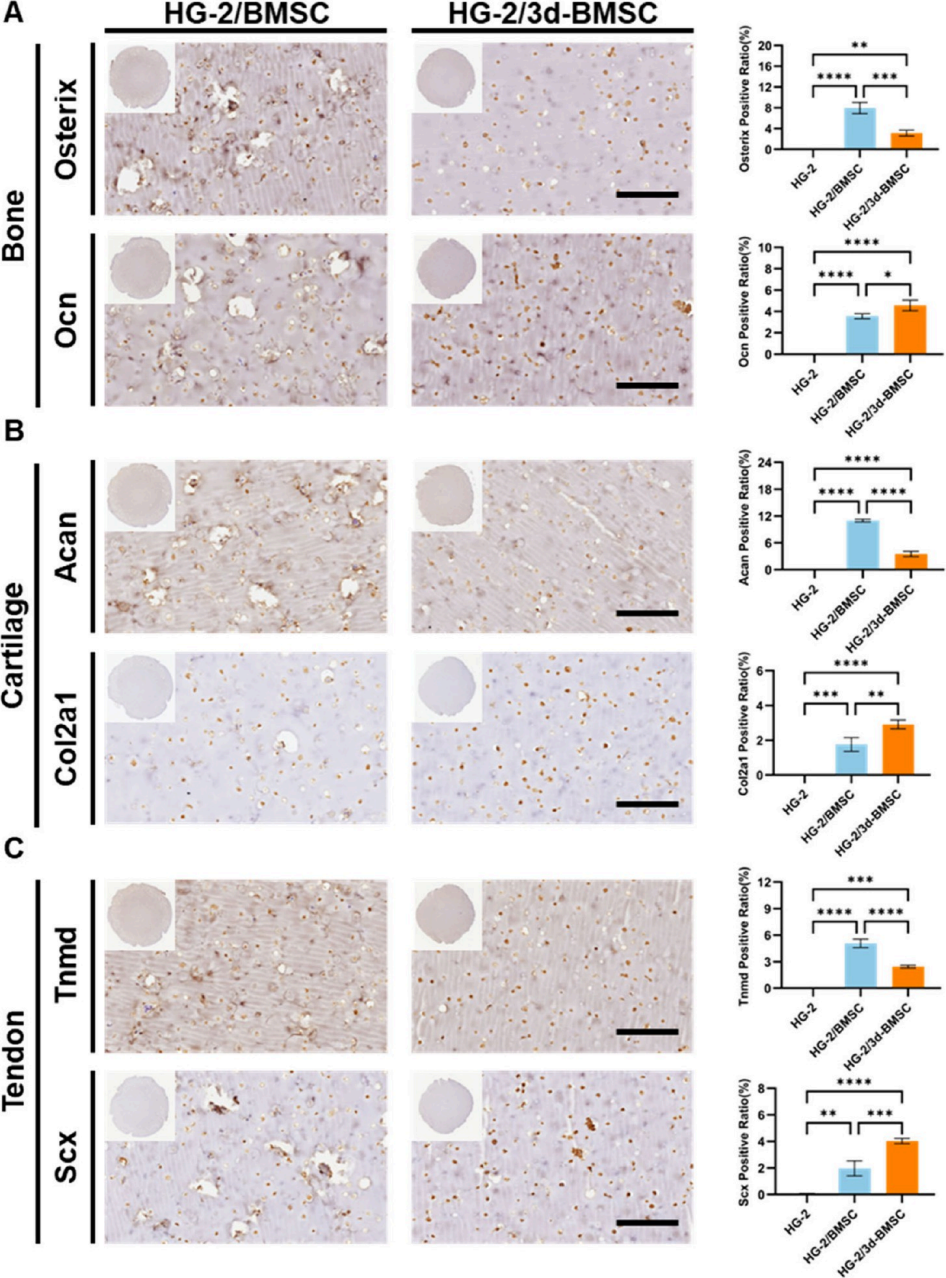
Immunohistochemical staining to evaluate multidirectional differentiation ability of obtained grafts in vivo. (A) Typical images and quantitative analysis of bone regeneration-related proteins at 6 weeks, one-way analysis of variance, one-tailed. (B) Typical images and quantitative analysis of cartilage regeneration-related proteins at 6 weeks, one-way analysis of variance, one-tailed. (C) Typical images and quantitative analysis of tendon regeneration-related proteins at 6 weeks, one-way analysis of variance, one-tailed. Scale bar = 200 μm. All data are expressed as mean ± SD (*n* = 3; *p < 0.05; **p < 0.01; ***p < 0.001; ****p < 0.0001). HG-2 group, ECM mimic hydrogel without BMSCs; HG-2/BMSC group, ECM mimic hydrogel with nonadherent BMSCs; HG-2/3d-BMSC group, ECM mimic hydrogel with adherent BMSCs.

We further investigated the biosafety of the hydrogel–cell complexes. The body weights of the mice were recorded during the experiment, and no significant difference was found (Figure S24). Moreover, blood biochemical tests also showed no significant metabolic abnormalities in all groups (Figure S25). There were no obvious pathological damages in representative H&E staining images of main organs shown in Figure S26, which confirmed the biosafety of prepared hydrogel–cell complexes as tissue regeneration implants.

The above results of in vivo experiments confirmed the potentials of HG-2/3d-BMSC organoid in accelerating multitissue regeneration. From the perspective of clinical transformation, the subcutaneous implantation model has the advantages of simple operation and strong repeatability, which can rapidly evaluate the biocompatibility and initial tissue induction ability of biomaterials at an early stage, and screen suitable materials and protocols for subsequent research.^48^ Moreover, the relatively single and confined environment of subcutaneous tissue provides a fundamental for evaluating the performance of organoids in their own evolution.^49^ However, the differential regulation of mechanical signal responses and complicated microenvironment of bone, cartilage, or tendon in situ also influence organoid differentiation.^50,51^ Thus, the absence of biomechanical loads specific to in situ tissues (such as cyclic pressure borne by articular cartilage) and tissue-specific inducing signals (such as periosteum-derived morphogenetic proteins) in subcutaneous models may lead to insufficient tissue specificity in organoid differentiation. More work is still needed in the future to confirm the regeneration ability of organoids in situ prior to clinical transformation.

### 3.5 ECM Mimic Hydrogel Derived Spatial Mechanical Stimulation Regulating Stem Cell Differentiation Fate via Yap/Tead4 Mechanotransduction Induced Kat7 Downregulation

Numerous studies have demonstrated that mechanical stimulation can promote Yap cytoplasm-to-nucleus translocation.^52–54^ Alberto et al. have highlighted that this phenomenon is achieved through the transmission of spatial mechanical stimulation from cell adhesion structures and intact cytoskeleton to the nucleus, resulting in nuclear pore opening.^55^ We have found that adhesion of stem cells to GelMA gels can increase Yap cytoplasm-to-nucleus translocation.^56^ Here in this work, we supposed that the adhesion that sits from the ECM mimic hydrogel could provide constant spatial mechanical stimulation, which might be the key factor of the accelerated and enhanced multidirectional differentiation ability of BMSCs.

In comparison with the HG-2/BMSC group, BMSCs in the HG-2/3d-BMSC group showed stronger Yap and nucleus co-localization, while the HG-2/3d-BMSC+CytoD group showed similar co-localization efficacy with the HG-2/BMSC group (Figure 7A,B and Figure S27). Cells within the HG-2/3d-BMSC group underwent cytoskeleton rearrangement induced by integrins-mediated adhesion to ECM mimic hydrogel. The assembled cytoskeleton fully sensed and efficiently transmitted spatial mechanical stimulation to the nucleus and subsequently promoted Yap nucleus translocation. The aforementioned phenomenon in the HG-2/3d-BMSC group could be inhibited by CytoD (HG-2/3d-BMSC+CytoD group) through the interruption of cytoskeletal rearrangement, which finally led to a decrease in the multidirectional differentiation capability of BMSCs to osteogenic, chondrogenic, tendon-genic cells (Figure 7C,D and Figures S28 and S29). Moreover, Yap protein was widely distributed in the whole BMSCs in the plate group and GelMA-coated group, and similarly Pearson’s correlation coefficient of Yap and nucleus also proved the consistency between these two groups (Figure S30A,B). The above results indicated that the adhesion induced spatial mechanical stimulation from the ECM mimic hydrogel rather than the ECM mimic hydrogel itself could increase Yap cytoplasmic-to-nucleus translocation.

**Figure 7:**
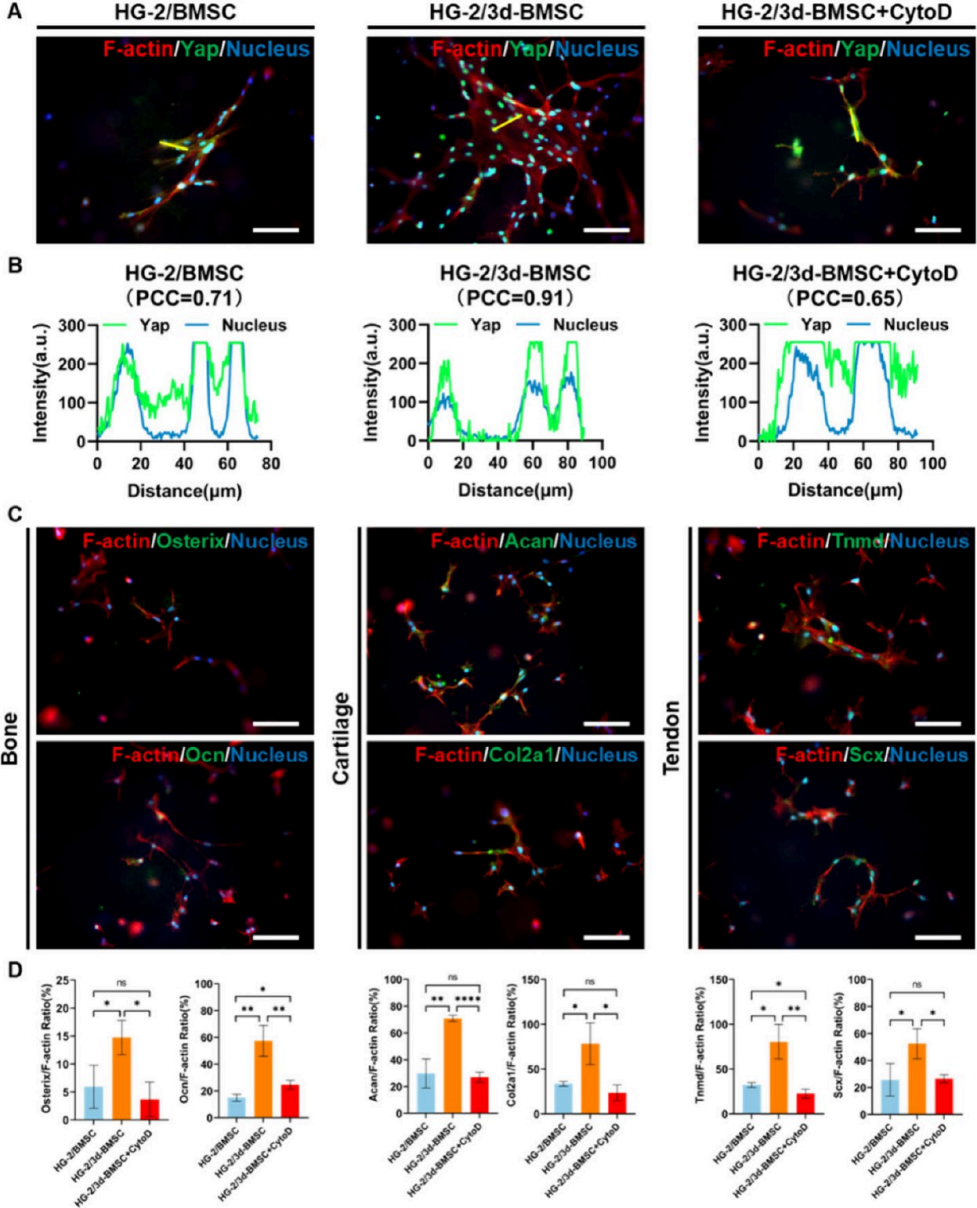
Cell adhesion-based spatial mechanical stimulation from ECM mimic hydrogel enhanced BMSCs multidirectional differentiation by increasing Yap cytoplasm-to-nucleus translocation. (A, B) Immunofluorescence staining and co-localization analysis of Yap protein at day 3 after osteogenic differentiation induction. The yellow line area is analyzed. PCC, Pearson’s correlation coefficient between Yap and nucleus. (C, D) Immunofluorescence images and semiquantitative analysis of typical bone regeneration-related proteins (Osterix, Ocn), cartilage regeneration-related proteins (Acan, Col2a1), and tendon regeneration-related proteins (Tnmd, Scx) expressions in HG-2/3d-BMSC+CytoD group at day 3 after osteogenic differentiation induction, one-way analysis of variance, one-tailed. Scale bar = 100 μm. All data are presented as mean ± SD (*n* = 3; *p < 0.05; **p < 0.01; ****p < 0.0001; ns means no significance). HG-2/BMSC group, ECM mimic hydrogel with nonadherent BMSCs; HG-2/3d-BMSC group, ECM mimic hydrogel with adherent BMSCs; HG-2/3d-BMSC+CytoD group, ECM mimic hydrogel with adherent BMSCs and treated with 0.2 μΜ CytoD.

In the Hippo signaling pathway, Tead is a critical mechanosensitive transcriptional regulator downstream of Yap,^57,58^ mRNA sequencing results also showed that the expression of TEAD4 gene in the Tead protein family is higher in HG-2/3d-BMSC group at all time points compared to the HG-2/BMSC group (Figure 8A and Figure S31), and the results were verified by a qRT-PCR experiment (Figure 8B). We further investigated the relationship between Tead4 and Yap protein translocation. CytoD was added to remove spatial mechanical stimulation induced cytoskeleton rearrangement, and Verteporfin was added to specifically inhibited the translocation of Yap protein from cytoplasm to nucleus. The qRT-PCR results indicated TEAD4 expression in the HG-2/3d-BMSC+CytoD group was significantly decreased, while the expression in the HG-2/3d-BMSC+Verteporfin group was similar to HG-2/3d-BMSC group, which confirmed that spatial mechanical stimulation induced cytoskeleton rearrangement, rather than Yap cytoplasm-to-nucleus translocation, could promote TEAD4 gene expression (Figure 8C). Immunofluorescence staining and co-localization analysis further confirmed the results, which also showed that high concentrations of Tead4 in the cytoplasm promoted its own nucleus transport (Figure 8D,E).

**Figure 8:**
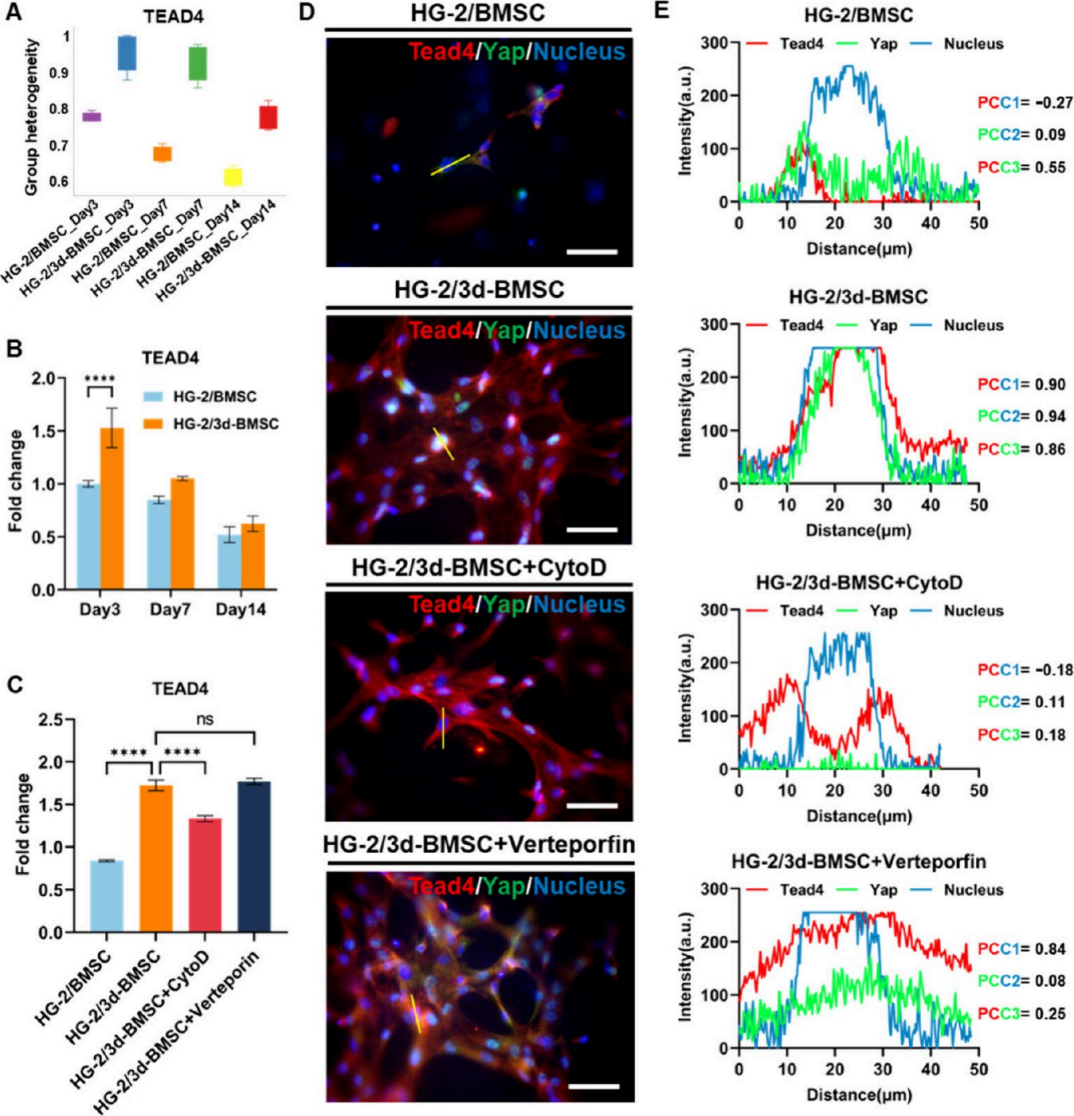
Cell adhesion-based spatial mechanical stimulation from ECM mimic hydrogel increasing the expression and cytoplasm-to-nucleus translocation of Tead4. (A, B) RNA-seq analysis the expression of TEAD4 gene in BMSCs in different groups at various times and confirmed by qRT-PCR, independent sample *t* test, one-tailed. (C) qRT-PCR result showing expression of TEAD4 in BMSCs with different inhibitors at day 3 after osteogenic differentiation induction, one-way analysis of variance, one-tailed. (D, E) Immunofluorescence staining and co-localization analysis of Tead4 protein, Yap protein, and nucleus of BMSCs in different groups at day 3 after osteogenic differentiation induction. The yellow line area is analyzed. Scale bar = 50 μm. PCC1, Pearson’s correlation coefficient between Tead4 and nucleus; PCC2, Pearson’s correlation coefficient between Yap and nucleus; PCC3, Pearson’s correlation coefficient between Tead4 and Yap. All data are presented as mean ± SD (*n* = 3; ***p < 0.001; ****p < 0.0001; ns means no significance). HG-2/BMSC group, ECM mimic hydrogel with nonadherent BMSCs; HG-2/3d-BMSC group, ECM mimic hydrogel with adherent BMSCs; HG-2/3d-BMSC+CytoD group, ECM mimic hydrogel with adherent BMSCs and treated with 0.2 μΜ CytoD; HG-2/3d-BMSC+Verteporfin group, ECM mimic hydrogel with adherent BMSCs and treated with 2 μΜ Verteporfin.

The Yap and Tead4 proteins can regulate transcription of DNA by forming complexes in the nucleus.^59^ Thus, co-localization of Yap and Tead4 proteins in the nucleus were essential. A strong co-localization relationship was proved among them in the HG-2/3d-BMSC group (Figure 8D,E and Figure S32). The nucleus translocations of Yap and Tead4 were both inhibited in the HG-2/3d-BMSC+CytoD group, while only Yap nucleus translocation was inhibited in the HG-2/3d-BMSC+Verteporfin group. These results further confirmed that the concentration-dependent nucleus translocation of Tead4 is triggered by spatial mechanical stimulation-induced cytoskeleton rearrangement rather than the Yap cytoplasm-to-nucleus translocation.

Co-localization of Yap and Tead4 in the nucleus could form a complex and bind to chromatin to regulate gene expression.^60,61^ The expressions of Yap/Tead4 downstream target genes CTGF and ANKRD1 were higher in the HG-2/3d-BMSC group at all time points (Figure S33A,B), and the expressions of CTGF and ANKRD1 were consistent with the nuclear localization of Yap (Figure 8D,E and Figure S33C,D), indicating that the function of Yap was activated. To further clarify the effects of spatial mechanical stimulation mediated Yap and Tead4 cytoplasm-to-nucleus translocation, we analyzed the mRNA sequencing results. It was found that the expression patterns of KAT7 gene in the HG-2/3d-BMSC group and HG-2/BMSC group were similar to the changes of TEAD4 expression at different times (Figure 9A). The results were verified by a qRT-PCR experiment (Figure 9B). The qRT-PCR results showed that KAT7 expression was significantly inhibited in the HG-2/3d-BMSC group, and the addition of CytoD (HG-2/3d-BMSC+CytoD group) and Verteporfin (HG-2/3d-BMSC+Verteporfin group) reversed the inhibition (Figure 9C). Immunofluorescence staining and semiquantitative analysis of Kat7 protein further confirmed the results (Figure 9D,E and Figure S34). Moreover, Kat7 protein expressions in the plate group and GelMA-coated group were also tested and no significant difference was founded (Figure S35A,B).

**Figure 9:**
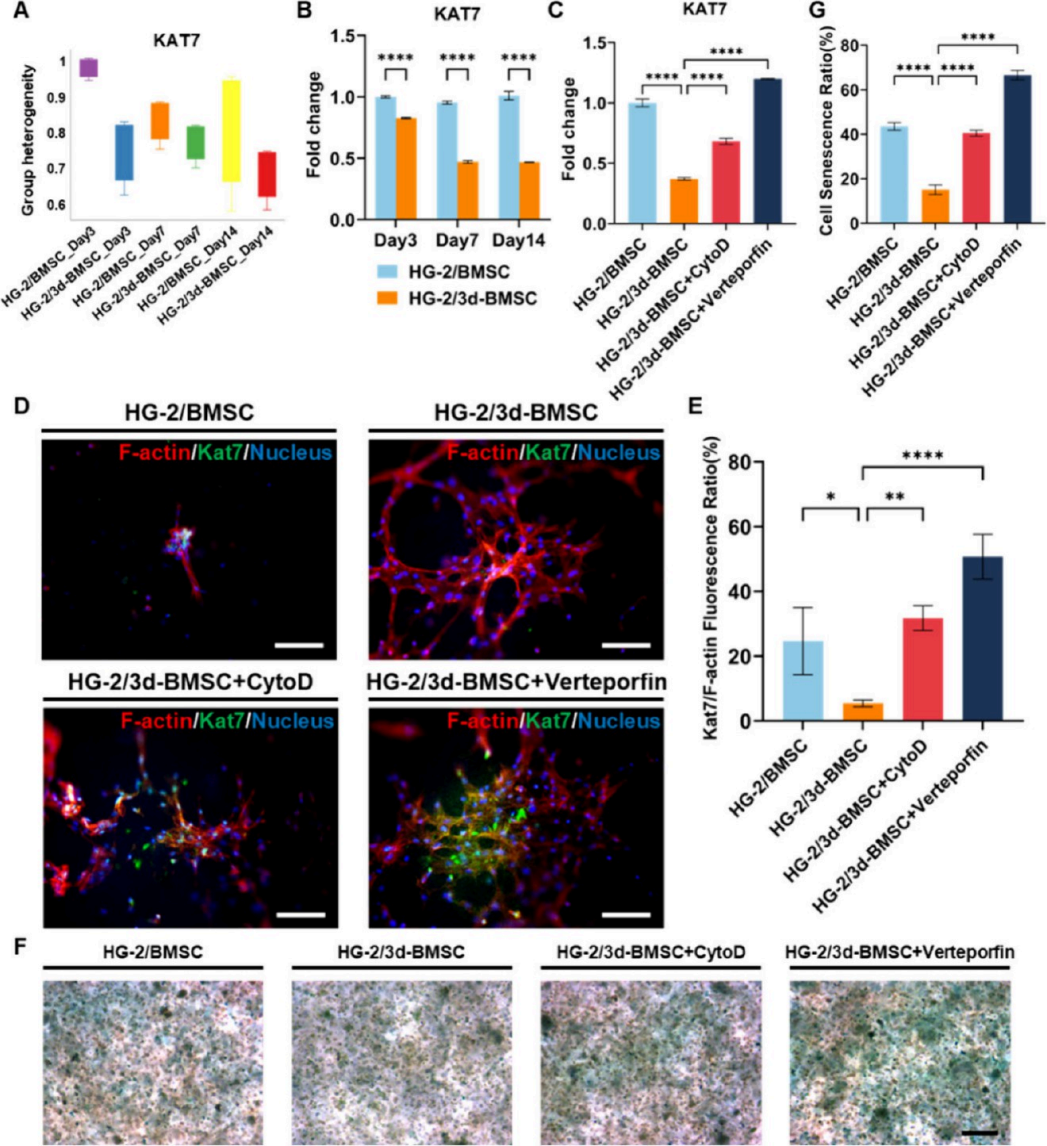
Cell adhesion-based spatial mechanical stimulation from ECM mimic hydrogel could delay BMSCs aging by suppressing Kat7 expression. (A, B) RNA-seq analysis showing the expression of KAT7 gene of BMSCs in different groups at various times after osteogenic differentiation induction and further confirmed by qRT-PCR, independent sample *t* test, one-tailed. (C) qRT-PCR result showed expression of KAT7 of BMSCs with different inhibitors at day 3 after osteogenic differentiation induction, one-way analysis of variance, one-tailed. (D, E) Immunofluorescence staining and semiquantitative analysis of Kat7 protein expression of BMSCs in different groups at Day 3 after osteogenic differentiation induction, one-way analysis of variance, one-tailed. Scale bar = 100 μm. (F, G) Assessment of cell senescence ratios in different groups with β-galactosidase staining at day 3 after osteogenic differentiation induction, one-way analysis of variance, one-tailed. Scale bar = 200 μm. All data are presented as mean ± SD (*n* = 3; *p < 0.05; **p < 0.01; ***p < 0.001; ****p < 0.0001; ns means no significance). HG-2/BMSC group, ECM mimic hydrogel with nonadherent BMSCs; HG-2/3d-BMSC group, ECM mimic hydrogel with adherent BMSCs; HG-2/3d-BMSC+CytoD group, ECM mimic hydrogel with adherent BMSCs and treated with 0.2 μΜ CytoD; HG-2/3d-BMSC+Verteporfin group, ECM mimic hydrogel with adherent BMSCs and treated with 2 μΜ Verteporfin.

A study has been reported in which KAT7 is a driver of cell senescence.^62^ Thus, we then evaluated the cell senescence of BMSCs in differently treated groups. The cell senescence ratio was significantly inhibited in the HG-2/3d-BMSC group, and the addition of CytoD (HG-2/3d-BMSC+CytoD group) and Verteporfin (HG-2/3d-BMSC+Verteporfin group) significantly increased this ratio (Figure 9F,G). Notably, BMSCs in the plate group and GelMA-coated group showed similar cell senescence ratios (Figure S35C,D), which indicated that the adhesion-induced spatial mechanical stimulation from ECM mimic hydrogel rather than ECM mimic hydrogel itself took part in regulating BMSCs fates.

Taken the above results together, we concluded that ECM mimic hydrogel could provide constant spatial mechanical stimulation to adherent stem cells, which not only enhanced the Yap translocation from cytoplasm to nucleus but also promoted Tead4 protein expression, and a high level of Tead4 in the cytoplasm further promoted nucleus import of itself. The Yap/Tead4 in nucleus further inhibited Kat7 cell senescence factor expression and, finally, enhanced and accelerated BMSCs multidirectional differentiation (Figure 10).

**Figure 10:**
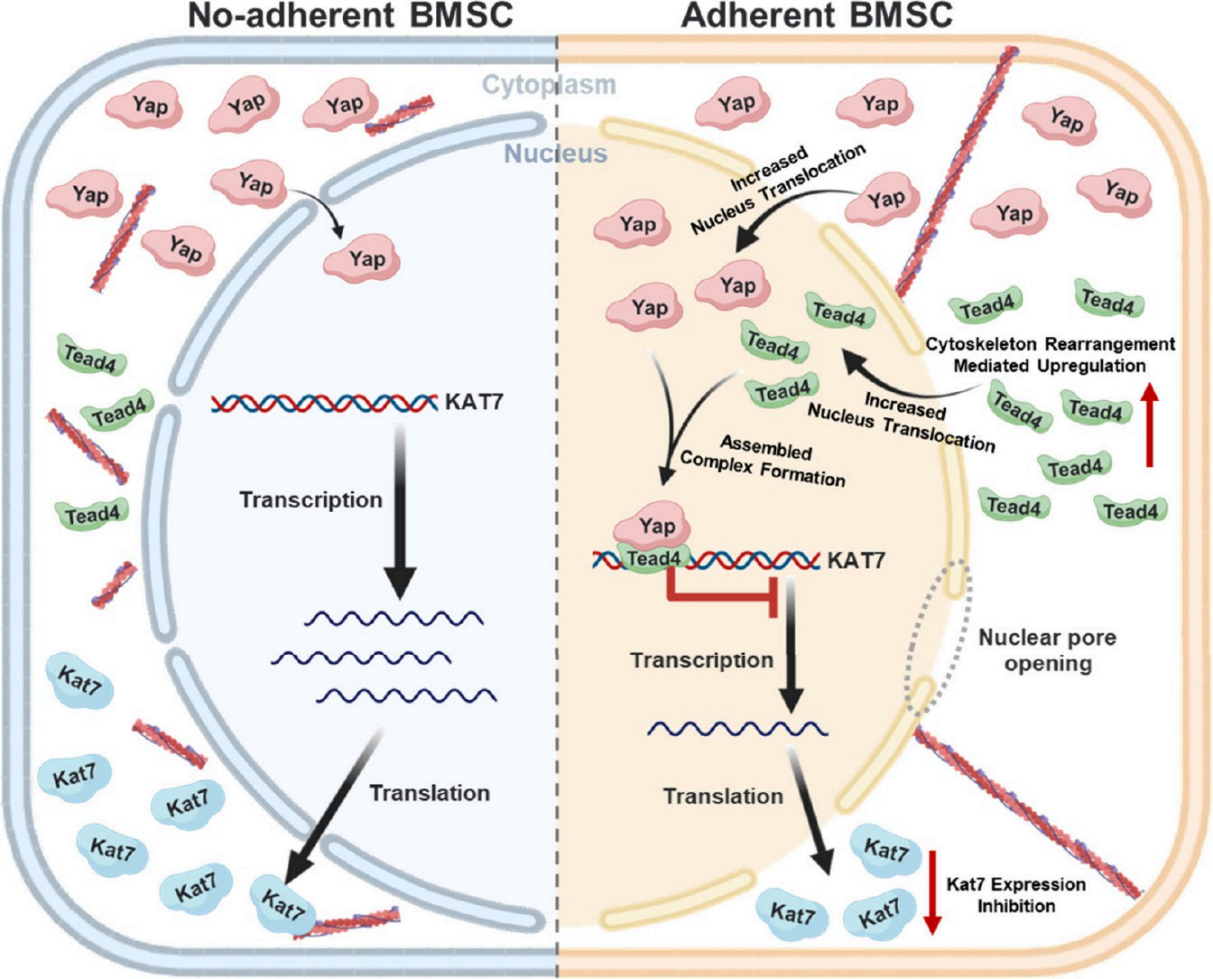
Diagram shows that the adhesion-based spatial mechanical stimulation regulates BMSCs fate via the Yap/Tead4 mechanotransduction mediated Kat7 downregulation. Adhesion-based spatial mechanical stimulation increases the expression of Tead4 and nucleus translocation of Yap, and a high level of Tead4 in the cytoplasm further promotes its nucleus translocation. Yap and Tead4 in nucleus combine to form a complex to suppress the level of Kat7 and finally enhance the potential of BMSCs for multidirectional differentiation.

This study also has several potential limitations: first, the epigenetic mechanism by which the Yap/Tead4 complex regulates Kat7 expression has not been fully elucidated; second, further investigations on expression patterns of other aging-related marker genes are scarce, such as P16, P21, and P53;^63–65^ third, the number of samples per time point in each group of animal experiments was insufficient; finally, the spatial mechanical stimuli in this study was static, and the effects of dynamic mechanical stimuli on cell differentiation and cell secretion profiles require further investigation.

## 4 Conclusions

In this study, we prepared a GelMA-based ECM mimic hydrogel to enhance multidirectional differentiation of the obtained organoid for potential repairment of multiple organs in the motor system. RNA-Seq combined with bioinformatics analysis and biological experiments in vitro demonstrated that adhesion-induced spatial mechanical stimulation from ECM mimic hydrogel could enhance and accelerate the multidirectional differentiation of BMSCs, and corresponding in vivo experiments further confirmed the multidirectional differentiation ability of the obtained HG-2/3d-BMSC organoid graft to bone, cartilage, and tendon. Further exploration proved that adhesion-based spatial mechanical stimulation enhanced the multidirectional differentiation ability of BMSCs by activating the Yap/Tead4 mechanotransduction mediated Kat7 downregulation. Our findings enrich the theory of biological materials in regulating stem cell fate and present promising organoid graft for repair of multiple organs in motor systems.

## Supplementary Material



## Notes

Biophysical characterization of hydrogels; adhesion and viability; two group organoids preparation process; RNA-seq quality analysis; KEGG and GO-BP enrichment analysis; various histochemistry staining and quantitative analysis results; biosafety of two obtained group organoids; immunofluorescence staining and co-localization analysis of Yap protein and nucleus in Plate group and GelMA-coated group; cell aging in Plate group and GelMA-coated group; sequence of primers; primary antibodies for immunofluorescence staining, etc. (PDF)
