# Supplementary material for: Hydrogel Enhanced Organoid Multidirectional Differentiation via Yap/Tead4 Mechanotransduction for Accelerated Tissue Regeneration
Source: ACS Appl Mater Interfaces. 2025 Jun 23;17(26):37601–16. doi: 10.1021/acsami.5c06161 (PMC12226991; doi:10.1021/acsami.5c06161)
Supplement: Supplementary file 1 [file am5c06161_si_001.pdf]

Supplementary Information

Hydrogel enhanced organoid multidirectional differentiation via Yap/Tead4 mechanotransduction for accelerated tissue regeneration

Peng Luo,‡ Yuning Cheng,‡ Yuwen Luo,‡ Nan Zhang, Jingjing Cao, Honggang Wang, Xieyuan Jiang, Qian Wang, Xinbao Wu, Yajun Liu, Jianping Mao, Xinhua Zhou, Jing-Jun Nie,* Dafu Chen**

Peng Luo, Yuning Cheng, Yuwen Luo, Nan Zhang, Jingjing Cao, Honggang Wang, Qian Wang, Jing-Jun Nie, Dafu Chen – Laboratory of Bone Tissue Engineering, Beijing Laboratory of Biomedical Materials, National Center for Orthopaedics, Beijing Research Institute of Traumatology and Orthopaedics, Beijing Jishuitan Hospital, Capital Medical University; Beijing, China

Xieyuan Jiang, Xinbao Wu – Department of Orthopedic Trauma, National Center for Orthopaedics, Beijing Jishuitan Hospital, Capital Medical University; Beijing, China

Yajun Liu, Jianping Mao – Department of Spine Surgery, National Center for Orthopaedics, Beijing Jishuitan Hospital, Capital Medical University; Beijing, China

Xinhua Zhou – Department of Orthopedic Surgery, National Center for Orthopaedics, Beijing Jishuitan Hospital, Capital Medical University; Beijing, China

‡These three authors contributed equally to this work.

* Corresponding Author. Email: chendafujst@126.com (D.-F.C.); niejingjun_jst@126.com (J.-J.N.); jxytrauma@163.com (X.J.)

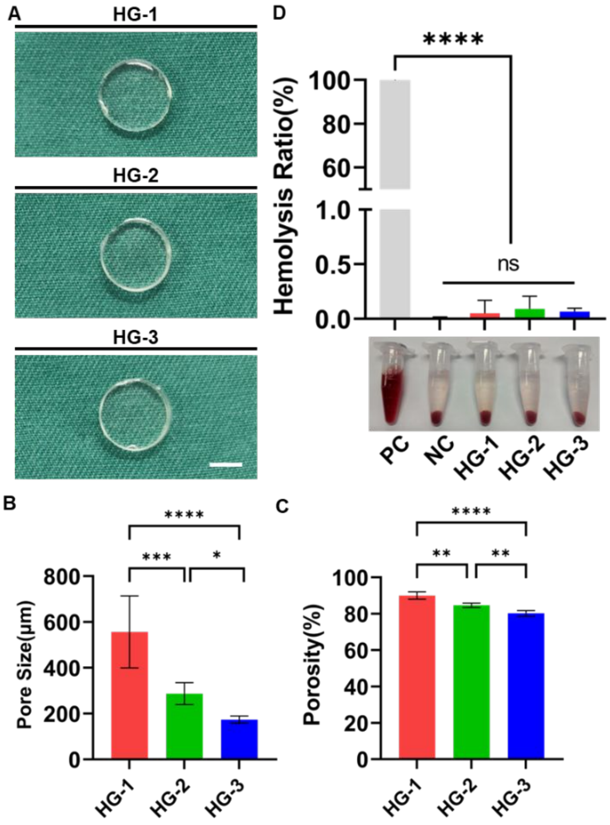

Figure S1. Biophysical characterization of the crosslinked GelMA-based ECM mimic hydrogel. A) Typical images showed the appearance of hydrogels with different mass volume fractions, Scale bar = 0.5 cm. B, C) Pore size and porosity of different hydrogels, one-way analysis of variance, one-tailed. D) Hemolysis assay showed the blood compatibility in vitro of the hydrogels, independent sample T test, two-tailed. All data are presented as mean±SD (n=4, **p*<0.05, ***p*<0.01, *****p*<0.0001, ns means no significance).

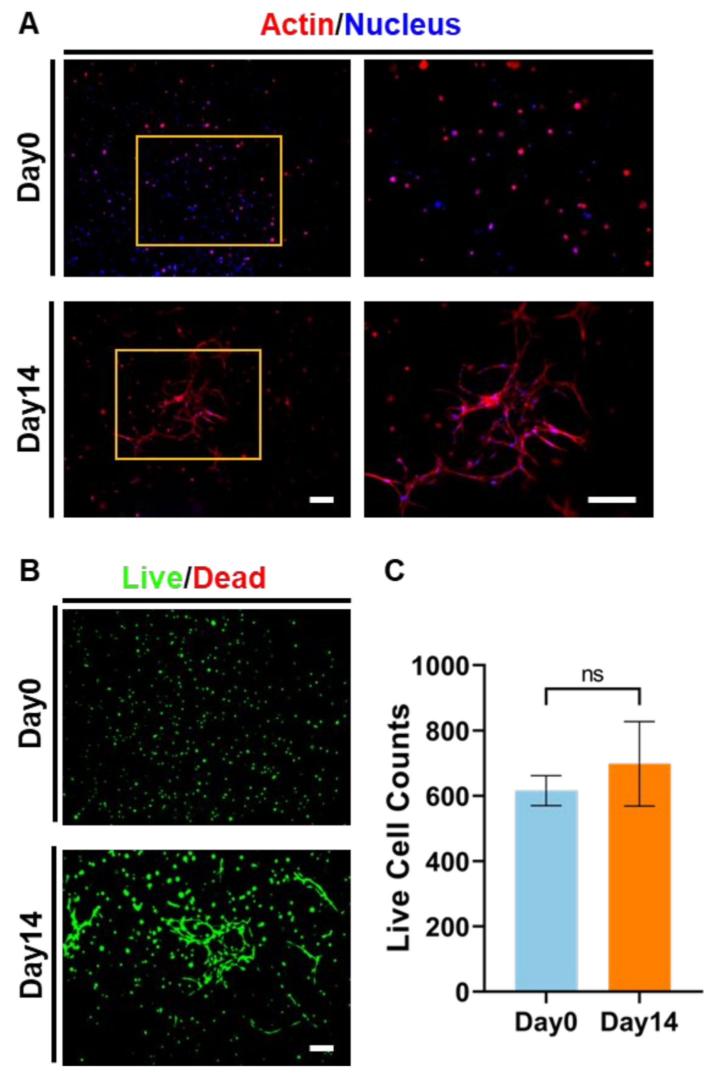

Figure S2. The cell adhesion and viability of BMSCs in GelMA-based ECM mimic hydrogel culturing in growth culture medium at Day0 and Day14. A) Immunofluorescence staining of cytoskeleton. B, C) Live/dead cell staining and live cell count of BMSCs in HG-2 hydrogel culturing in growth culture medium at Day0 and Day14, independent sample T test, two-tailed. Scale bar = 100 μm. All data are presented as mean±SD (n=3, ns means no significance).

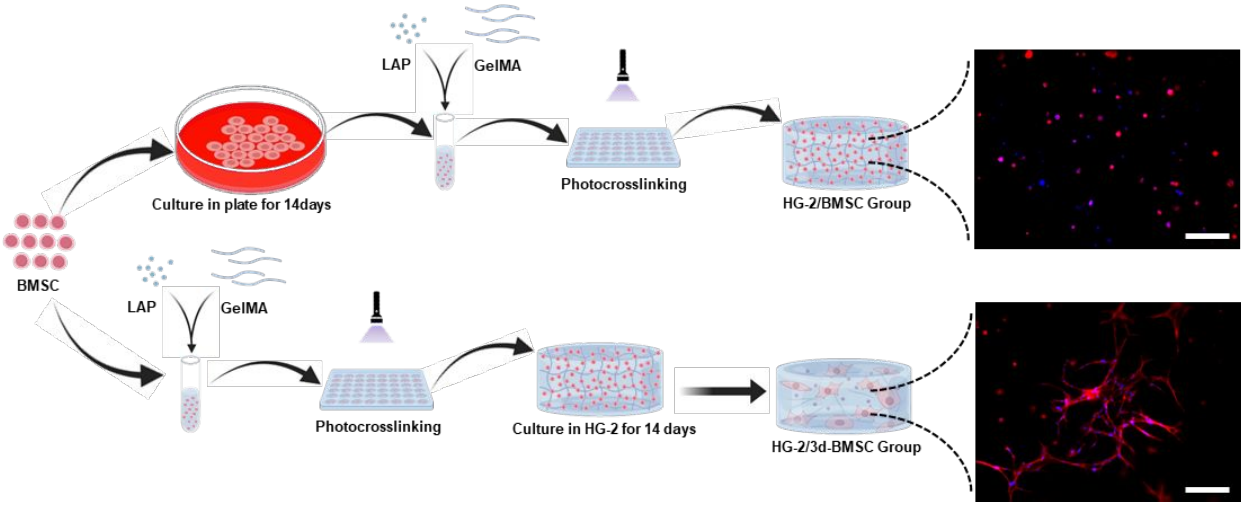

Figure S3. HG-2/3d-BMSC group and HG-2/BMSC group organoids preparation process. BMSCs cultured in dishes were collected and divided into two parts. One part of the BMSCs was resuspended and added into plate, followed by culturing in growth culture medium for 14 days. The cells were then collected and resuspended with HG-2 GelMA mixture at the density of 4×10^6 cells/mL to get BMSCs loaded HG-2 hydrogels, and cultured with osteogenic differentiation induction medium to obtain the type Ⅰ organoid (termed as HG-2/BMSC group). The other part of the cells was directly resuspended with HG-2 GelMA mixture at the density of 4×10^6 cells/mL, followed by culturing in growth culture medium for 14 days to get HG-2 hydrogels with fully attached and extended three-dimensional BMSCs, and then cultured with osteogenic differentiation induction medium to obtain the type Ⅱ organoid (termed as HG-2/3d-BMSC group). Scale bar = 100 μm.

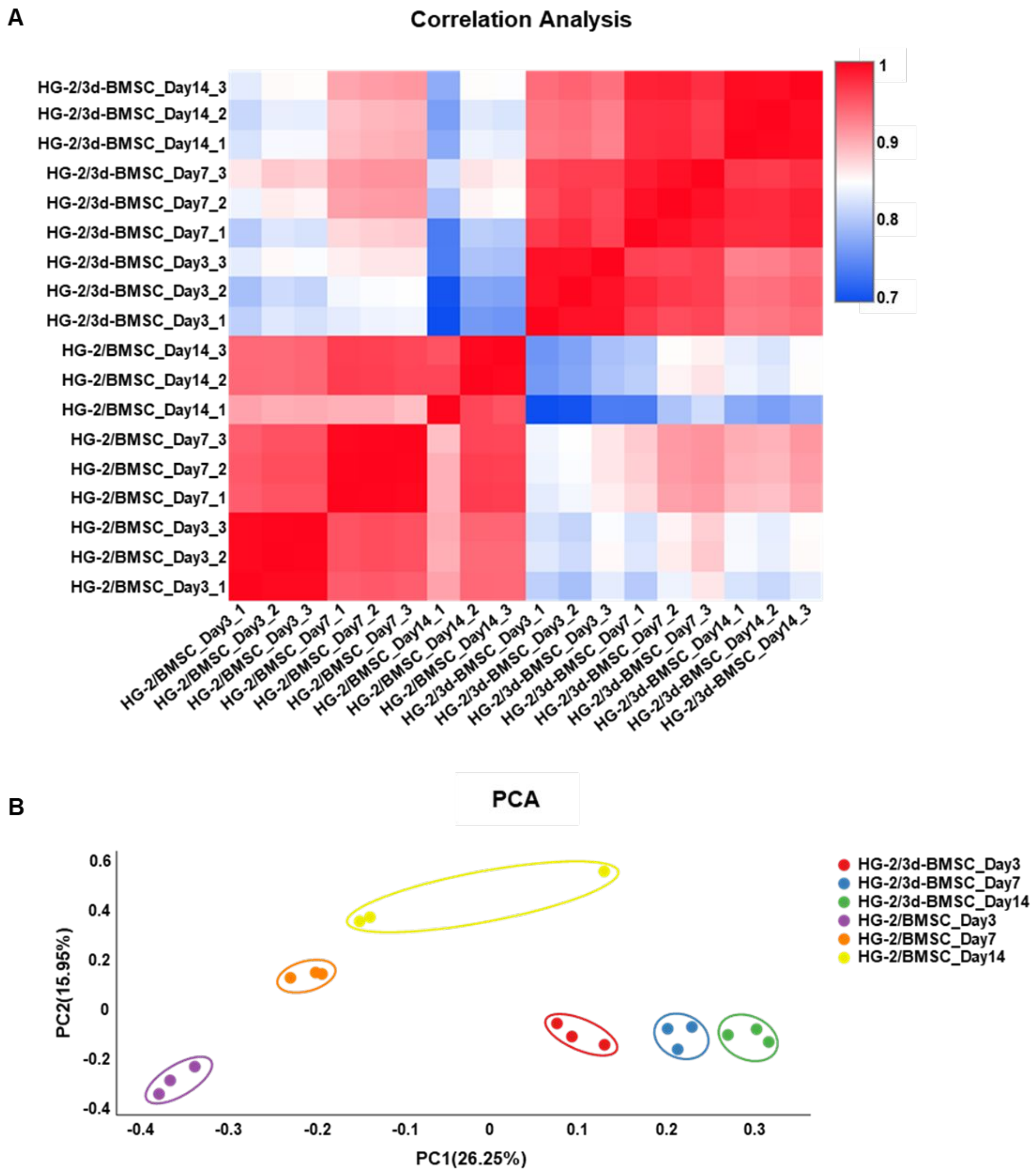

Figure S4. RNA-seq quality analysis. A) Correlation analysis of BMSCs RNA in different groups after osteogenic differentiation induction for different times (n=3). B) Principal component analysis of BMSCs RNA in different groups after osteogenic differentiation induction for different times (n=3). HG-2/BMSC group, ECM mimic hydrogel with non-adherent BMSCs; HG-2/3d-BMSC group, ECM mimic hydrogel with adherent BMSCs.

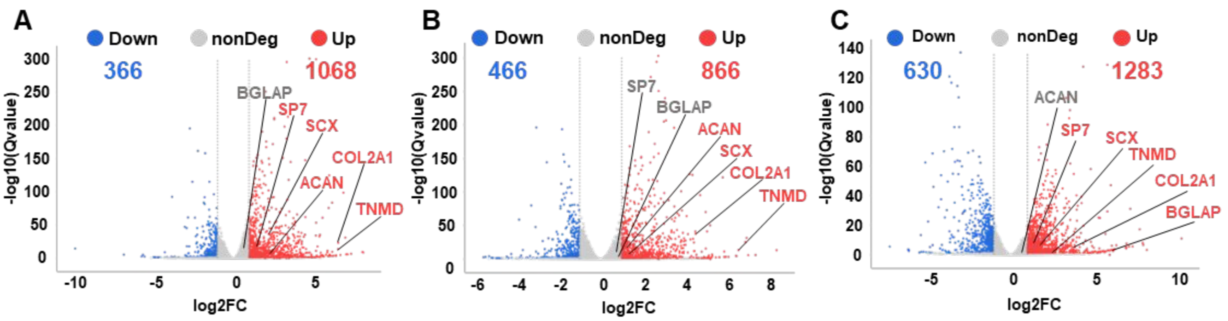

Figure S5. Volcano plots and representative genes annotation showed gene expression of BMSCs after osteogenic differentiation induction for 3 days A), 7 days B) and 14 days C) (HG-2/3d-BMSC vs. HG-2/BMSC. log2 fold change >1, < −1; $p < 0.05$; n=3). HG-2/BMSC group, ECM mimic hydrogel with non-adherent BMSCs; HG-2/3d-BMSC group, ECM mimic hydrogel with adherent BMSCs.

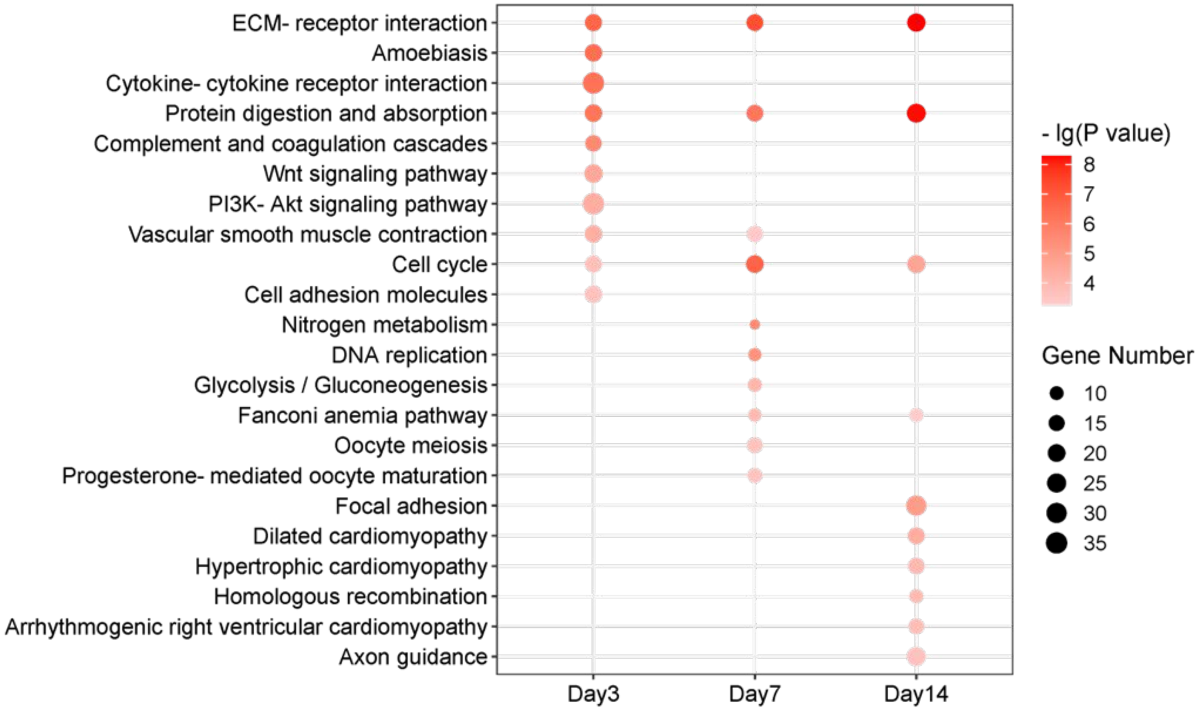

Figure S6. KEGG enrichment analysis of up-regulated differential genes of BMSCs after osteogenic differentiation induction for different times (HG-2/3d-BMSC vs HG-2/BMSC). HG-2/BMSC group, ECM mimic hydrogel with non-adherent BMSCs; HG-2/3d-BMSC group, ECM mimic hydrogel with adherent BMSCs.

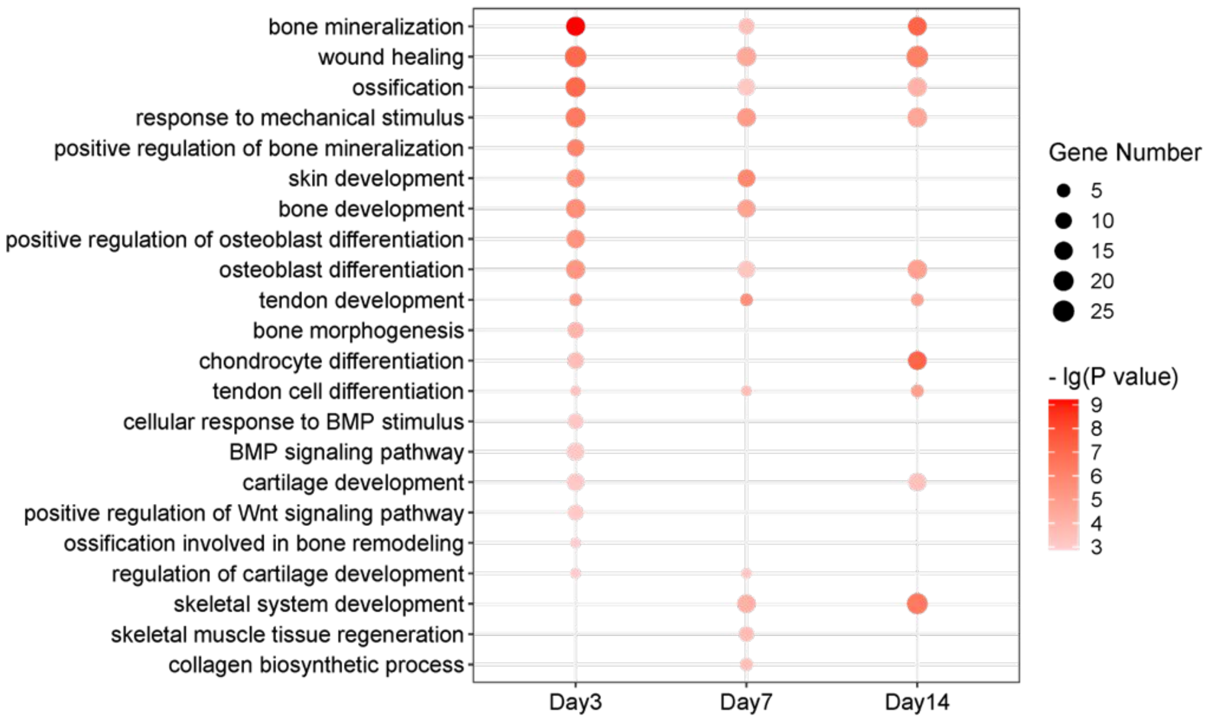

Figure S7. GO-BP enrichment analysis of up-regulated differential genes of BMSCs after osteogenic differentiation induction for different times (HG-2/3d-BMSC vs HG-2/BMSC). HG-2/BMSC group, ECM mimic hydrogel with non-adherent BMSCs; HG-2/3d-BMSC group, ECM mimic hydrogel with adherent BMSCs.

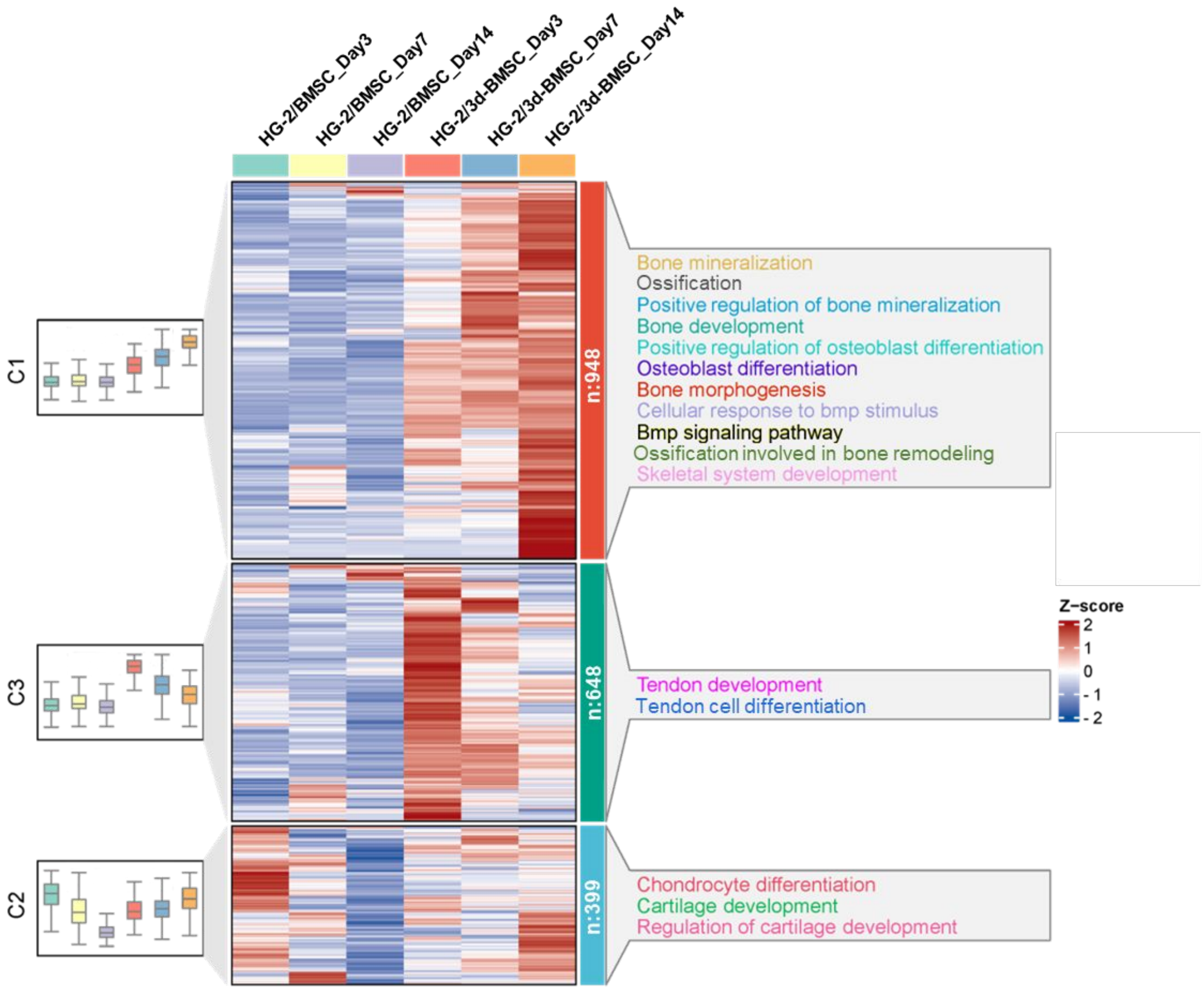

Figure S8. Time trend transcriptome analysis showed the pathway enrichment changes of bone, cartilage and tendon regeneration after osteogenic differentiation induction for 3, 7 and 14 days. HG-2/BMSC group, ECM mimic hydrogel with non-adherent BMSCs; HG-2/3d-BMSC group, ECM mimic hydrogel with adherent BMSCs.

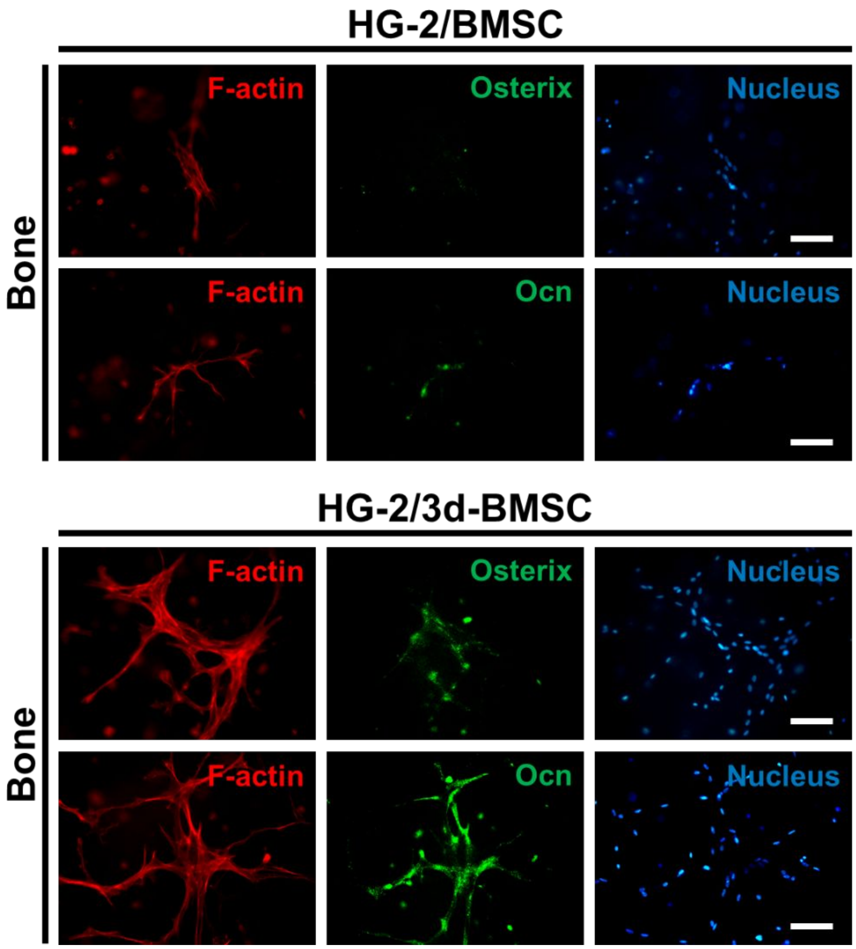

Figure S9. Immunofluorescence images of typical bone regeneration-related proteins (Osterix, Ocn) of BMSCs in different groups at Day3 after induction (Scale bar = 100 μm). HG-2/BMSC group, ECM mimic hydrogel with non-adherent BMSCs; HG-2/3d-BMSC group, ECM mimic hydrogel with adherent BMSCs.

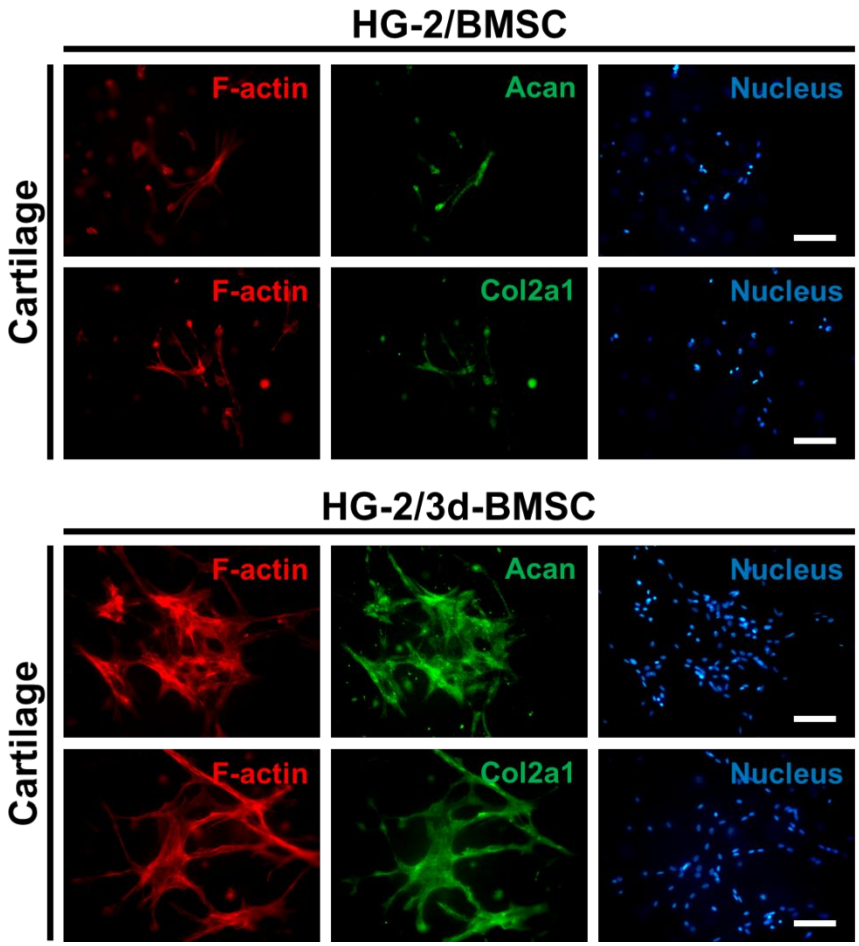

Figure S10. Immunofluorescence images of typical cartilage regeneration-related proteins (Acan, Col2a1) of BMSCs in different groups at Day3 after induction (Scale bar = 100 μm). HG-2/BMSC group, ECM mimic hydrogel with non-adherent BMSCs; HG-2/3d-BMSC group, ECM mimic hydrogel with adherent BMSCs.

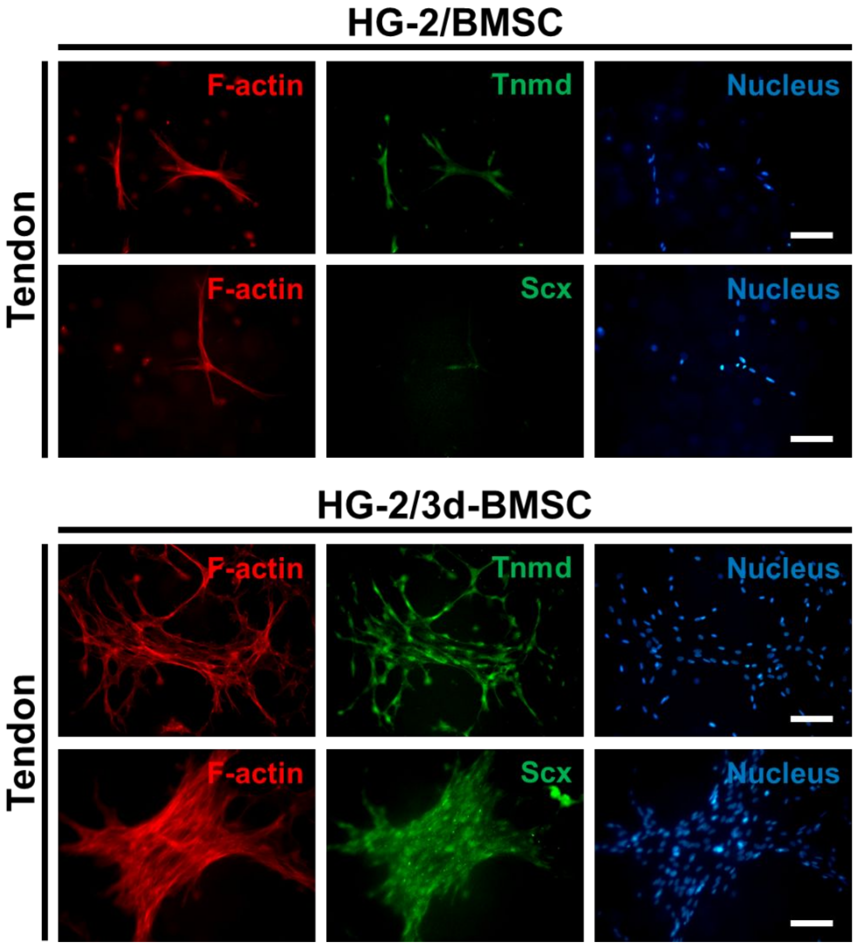

Figure S11. Immunofluorescence images of typical tendon regeneration-related proteins (Tnmd, Scx) of BMSCs in different groups at Day3 after induction (Scale bar = 100 μm). HG-2/BMSC group, ECM mimic hydrogel with non-adherent BMSCs; HG-2/3d-BMSC group, ECM mimic hydrogel with adherent BMSCs.

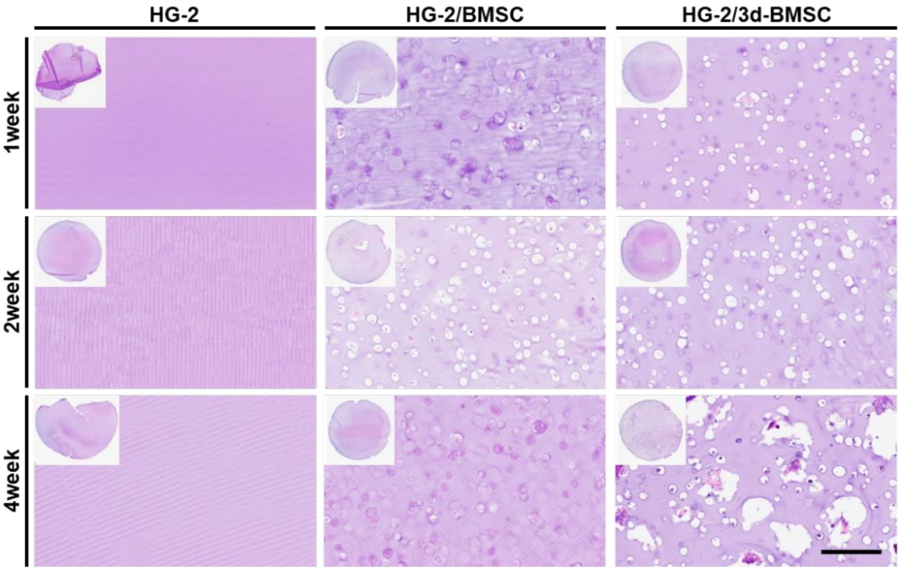

Figure S12. H&E staining showed new tissue generation of different groups after implantation of 1, 2, and 4 weeks in vivo. Scale bar = 200 μm. HG-2 group, ECM mimic hydrogel without BMSCs; HG-2/BMSC group, ECM mimic hydrogel with non-adherent BMSCs; HG-2/3d-BMSC group, ECM mimic hydrogel with adherent BMSCs.

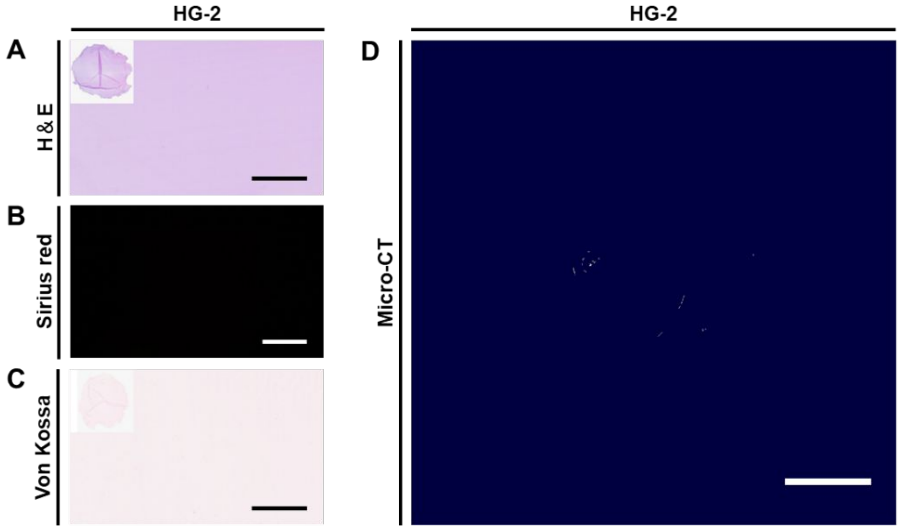

Figure S13. In vivo multidirectional differentiation ability of obtained grafts after osteogenic differentiation induction for 14 days. A) H&E staining showed new tissue generation of HG-2 group after 6 weeks, Scale bar = 200 μm. B) Sirius red staining (under polarized light microscope) showed collagen fiber formation of HG-2 group after 6 weeks, Scale bar = 50 μm. C) Von Kossa staining showed the calcium salt deposition of HG-2 group after 6 weeks, Scale bar = 200 μm. D) Micro-CT scan reconstruction provided the mineralization degrees of HG-2 group after 6 weeks, Scale bar = 200 mm. HG-2 group, ECM mimic hydrogel without BMSCs.

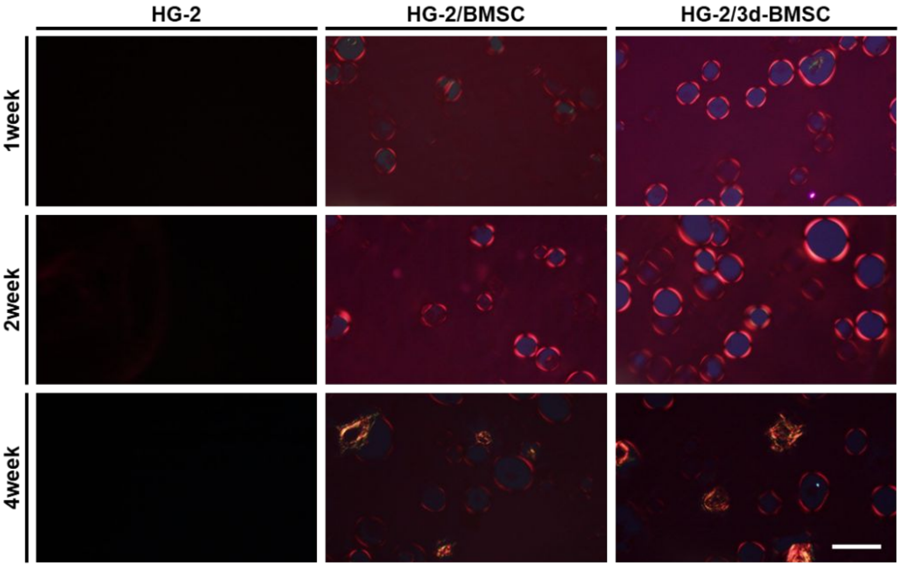

Figure S14. Representative Sirius red staining showed collagen fiber formation in different groups after implantation of 1, 2, and 4 weeks in vivo (under polarized light microscope). Scale bar = 50 μm. HG-2 group, ECM mimic hydrogel without BMSCs; HG-2/BMSC group, ECM mimic hydrogel with non-adherent BMSCs; HG-2/3d-BMSC group, ECM mimic hydrogel with adherent BMSCs.

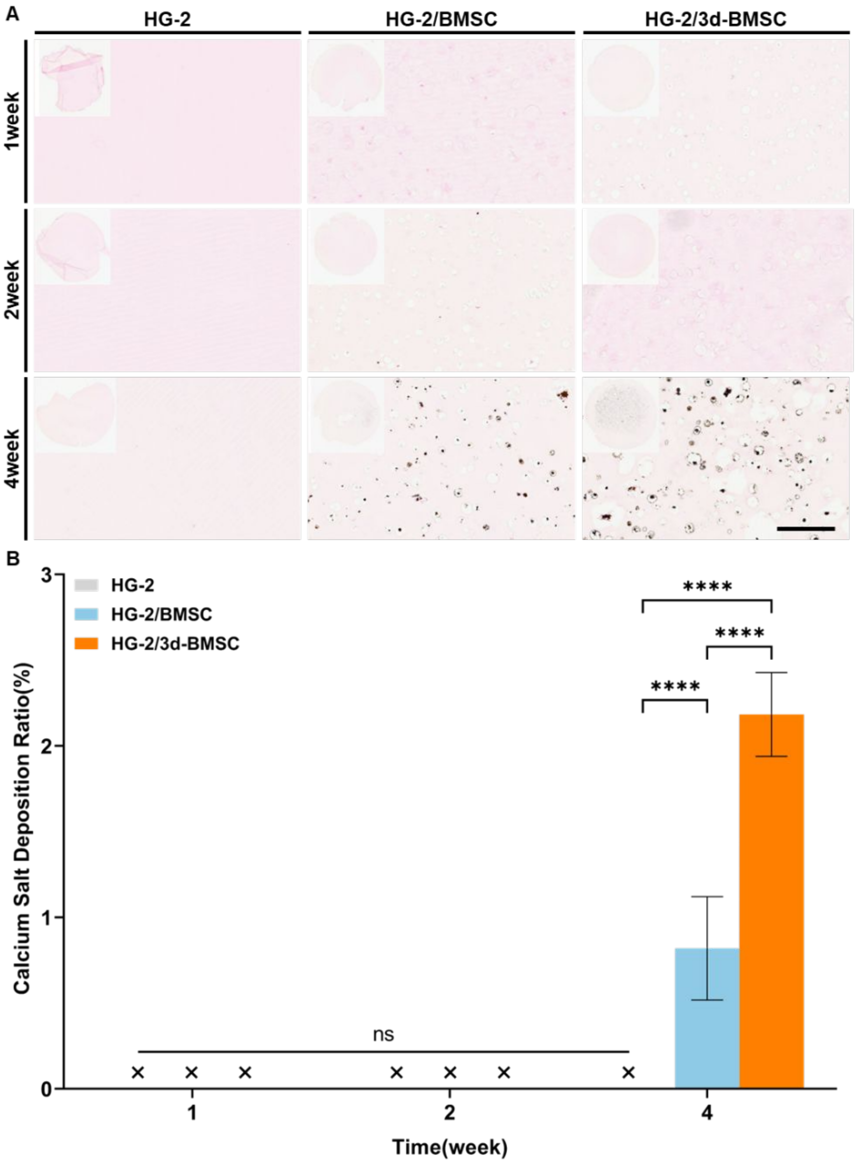

Figure S15. Von Kossa staining images A) and quantitative analysis results B) showed the calcium salt deposition of different groups after 1, 2, and 4 weeks in vivo, one-way analysis of variance, one-tailed. Scale bar = 200 μm. All data are expressed as mean ± SD (n=3, ****$p < 0.0001$, ns means no significance). HG-2 group, ECM mimic hydrogel without BMSCs; HG-2/BMSC group, ECM mimic hydrogel with non-adherent BMSCs; HG-2/3d-BMSC group, ECM mimic hydrogel with adherent BMSCs.

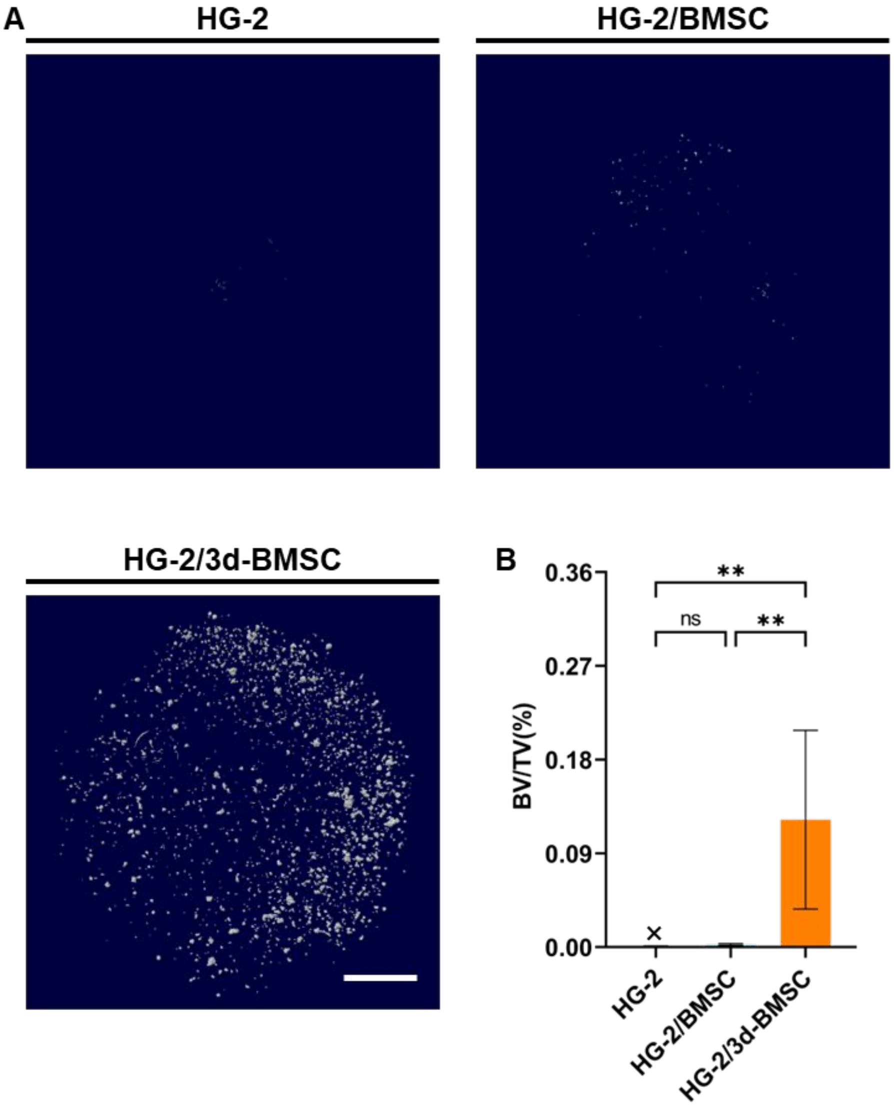

Figure S16. Micro-CT scan reconstruction A) and quantitative analysis results B) provided the mineralization ratio of different groups after 4 weeks in vivo, one-way analysis of variance, one-tailed. Scale bar = 200 mm. All data are expressed as mean ± SD (n=3, ***p* < 0.01, ns means no significance). HG-2 group, ECM mimic hydrogel without BMSCs; HG-2/BMSC group, ECM mimic hydrogel with non-adherent BMSCs; HG-2/3d-BMSC group, ECM mimic hydrogel with adherent BMSCs.

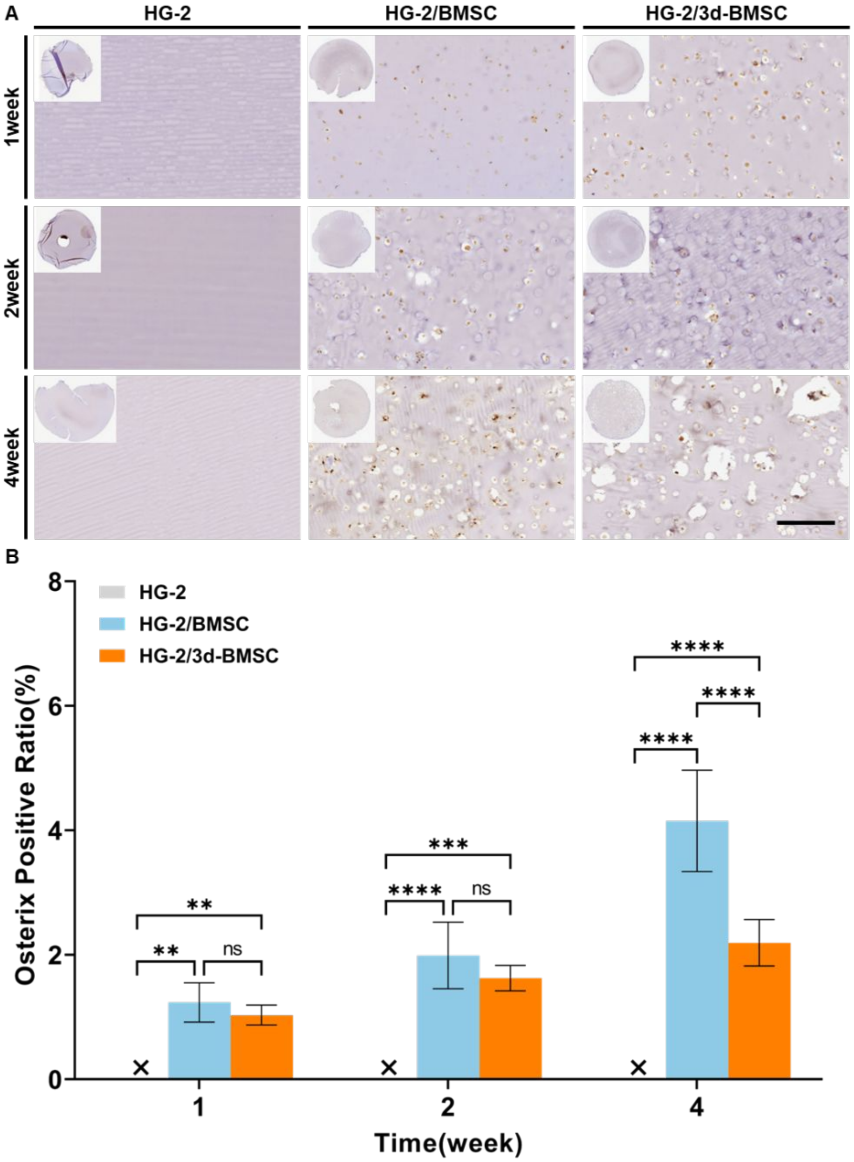

Figure S17. Typical Immunohistochemical staining images A) and quantitative analysis results B) of Osterix proteins of different groups at 1, 2, and 4 weeks in vivo, one-way analysis of variance, one-tailed. Scale bar = 200 µm. All data are expressed as mean ± SD (n=3, ***p* < 0.01, ****p* < 0.001, *****p* < 0.0001, ns means no significance). HG-2 group, ECM mimic hydrogel without BMSCs; HG-2/BMSC group, ECM mimic hydrogel with non-adherent BMSCs; HG-2/3d-BMSC group, ECM mimic hydrogel with adherent BMSCs.

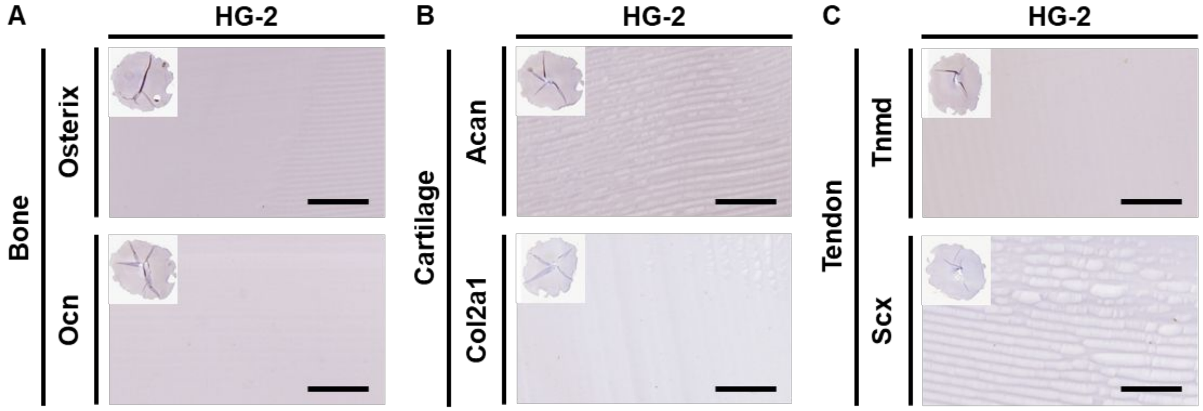

Figure S18. Immunohistochemical staining to evaluate multidirectional differentiation ability of obtained HG-2 group grafts in vivo. A) Typical images of bone regeneration-related proteins at 6 weeks. B) Typical images of cartilage regeneration-related proteins at 6 weeks. C) Typical images of tendon regeneration-related proteins at 6 weeks. HG-2 group, ECM mimic hydrogel without BMSCs.

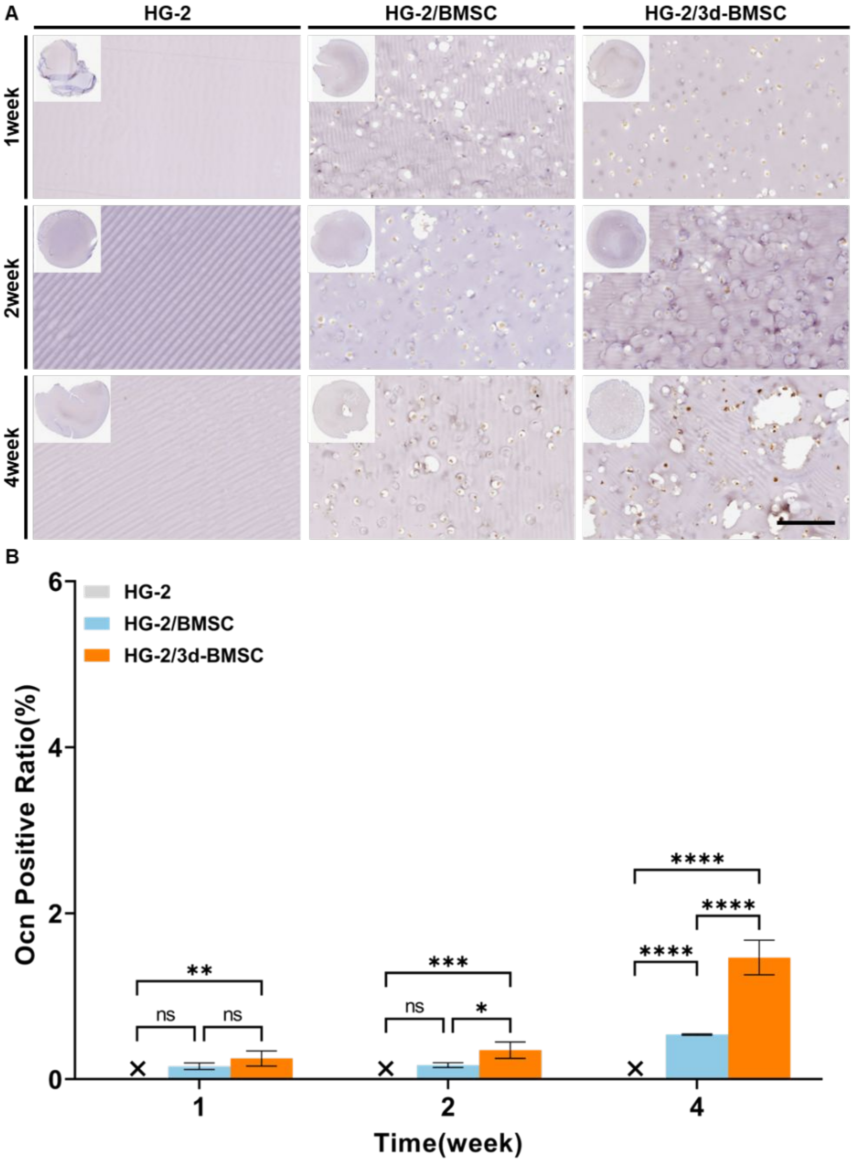

Figure S19. Typical Immunohistochemical staining images A) and quantitative analysis results B) of Ocn proteins of different groups at 1, 2, and 4 weeks in vivo, one-way analysis of variance, one-tailed**.** Scale bar = 200 μm. All data are expressed as mean ± SD (n=3, **p* < 0.05, ***p* < 0.01, ****p* < 0.001, *****p* < 0.0001, ns means no significance). HG-2 group, ECM mimic hydrogel without BMSCs; HG-2/BMSC group, ECM mimic hydrogel with non-adherent BMSCs; HG-2/3d-BMSC group, ECM mimic hydrogel with adherent BMSCs.

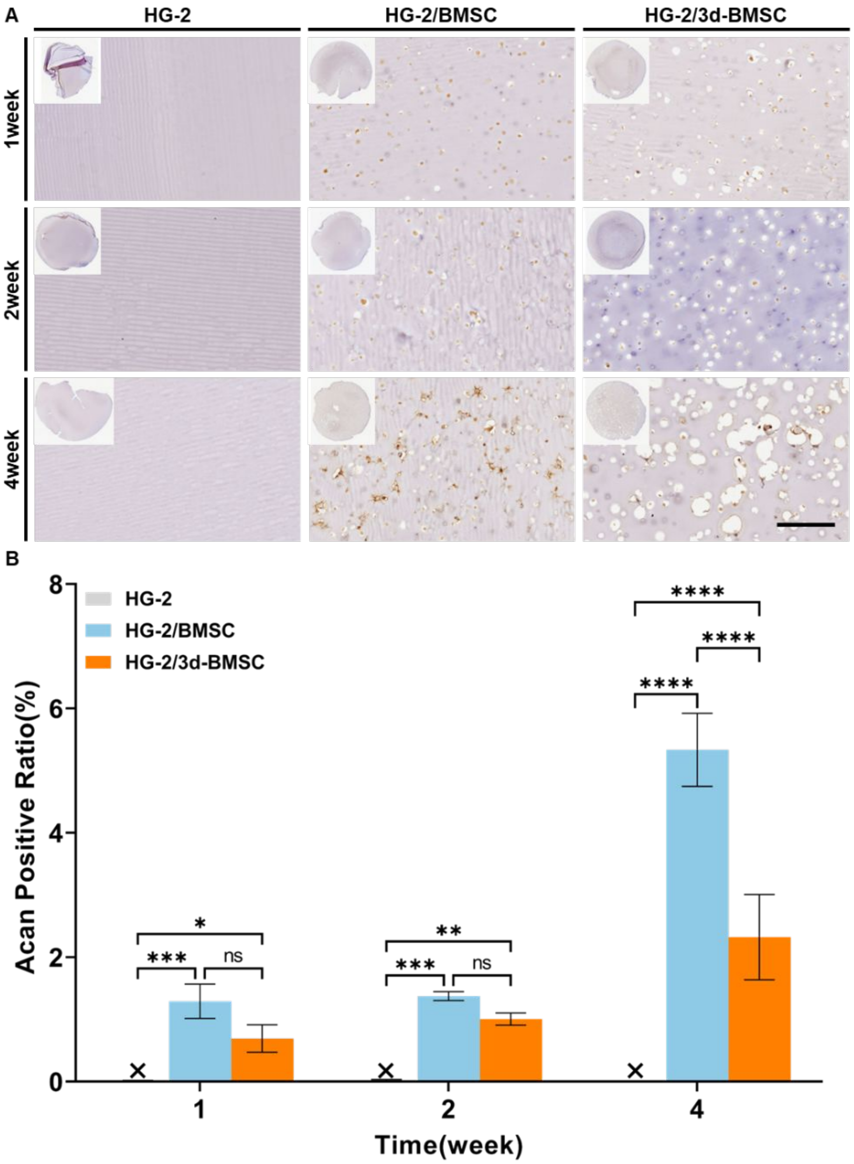

Figure S20. Typical Immunohistochemical staining images A) and quantitative analysis results B) of Acan proteins of different groups at 1, 2, and 4 weeks in vivo, one-way analysis of variance, one-tailed**.** Scale bar = 200 μm. All data are expressed as mean ± SD (n=3, $*p < 0.05$, $**p < 0.01$, $***p < 0.001$, $****p < 0.0001$, ns means no significance). HG-2 group, ECM mimic hydrogel without BMSCs; HG-2/BMSC group, ECM mimic hydrogel with non-adherent BMSCs; HG-2/3d-BMSC group, ECM mimic hydrogel with adherent BMSCs.

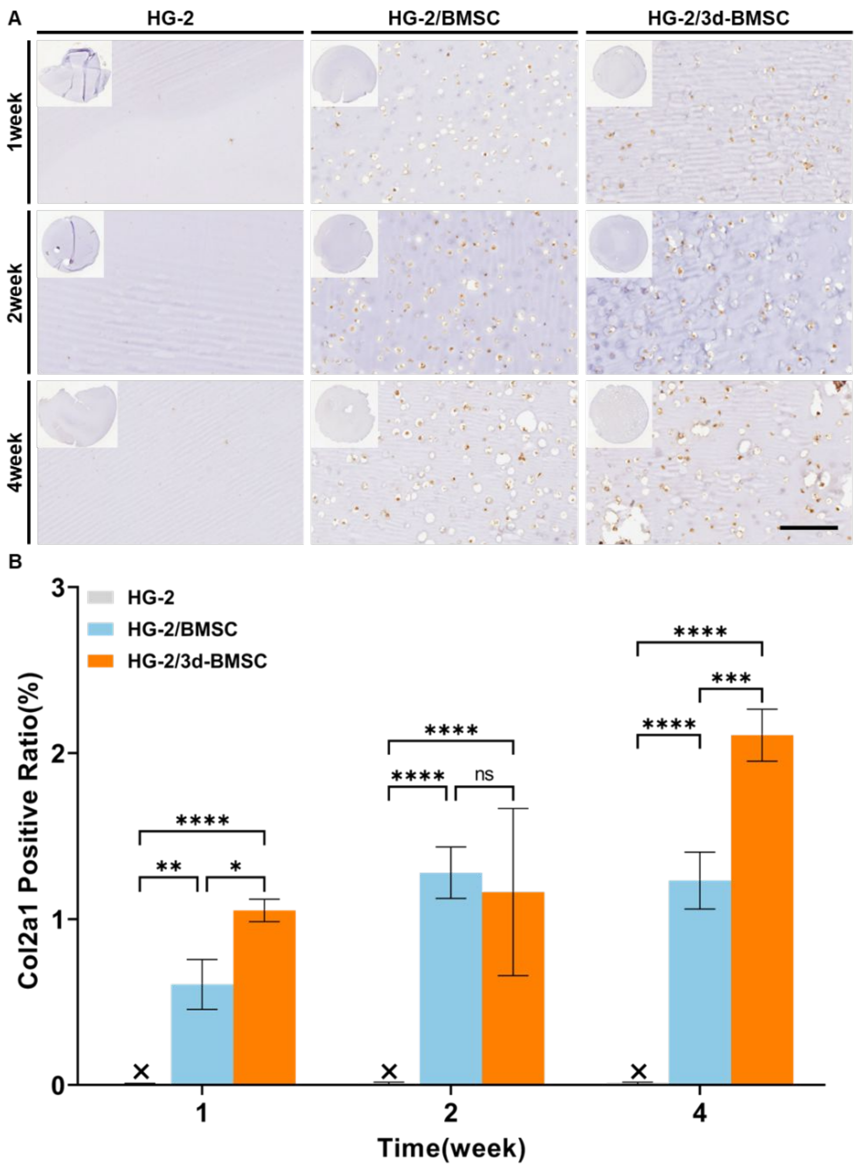

Figure S21. Typical Immunohistochemical staining images A) and quantitative analysis results B) of Col2a1 proteins of different groups at 1, 2, and 4 weeks in vivo, one-way analysis of variance, one-tailed**.** Scale bar = 200 μm. All data are expressed as mean ± SD (n=3, *$p < 0.05$, **$p < 0.01$, ***$p < 0.001$, ****$p < 0.0001$, ns means no significance). HG-2 group, ECM mimic hydrogel without BMSCs; HG-2/BMSC group, ECM mimic hydrogel with non-adherent BMSCs; HG-2/3d-BMSC group, ECM mimic hydrogel with adherent BMSCs.

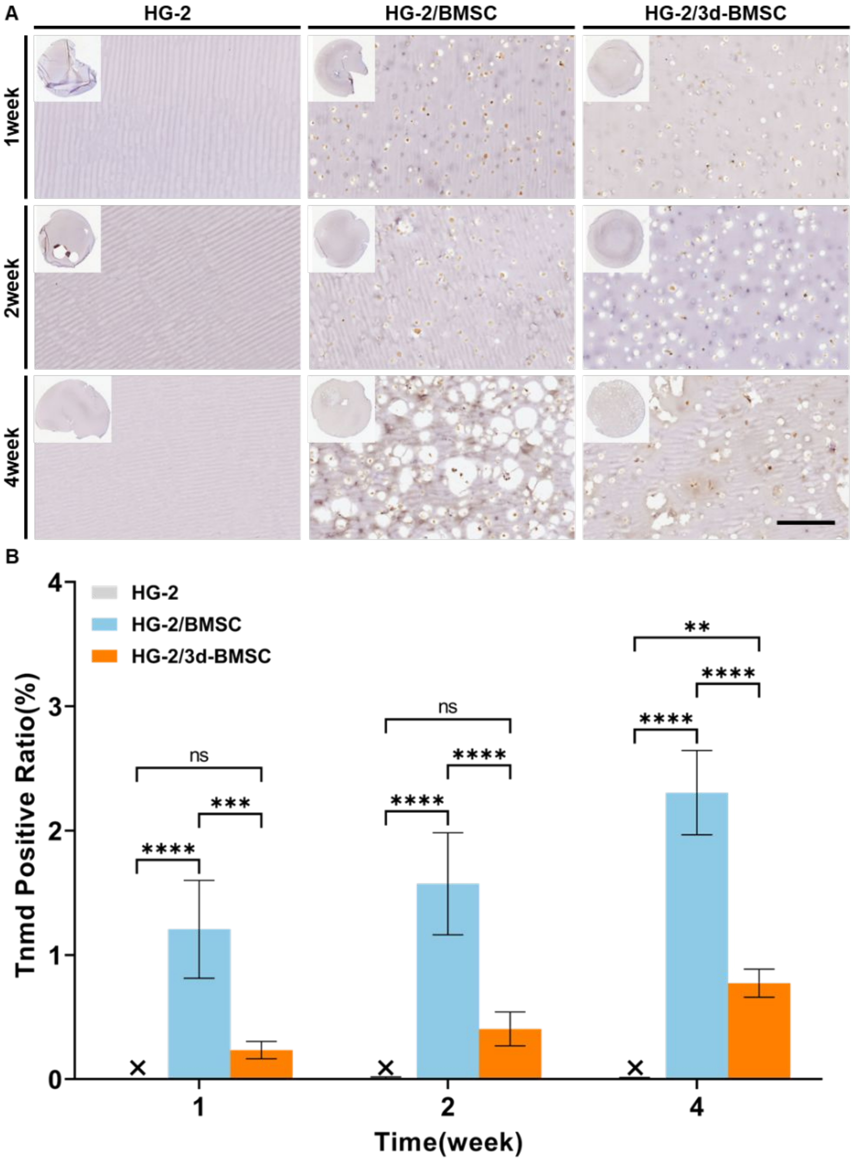

Figure S22. Typical Immunohistochemical staining images A) and quantitative analysis results B) of Tnmd proteins of different groups at 1, 2, and 4 weeks in vivo, one-way analysis of variance, one-tailed**.** Scale bar = 200 μm. All data are expressed as mean ± SD (n=3, **$p < 0.01$, ***$p < 0.001$, ****$p < 0.0001$, ns means no significance). HG-2 group, ECM mimic hydrogel without BMSCs; HG-2/BMSC group, ECM mimic hydrogel with non-adherent BMSCs; HG-2/3d-BMSC group, ECM mimic hydrogel with adherent BMSCs.

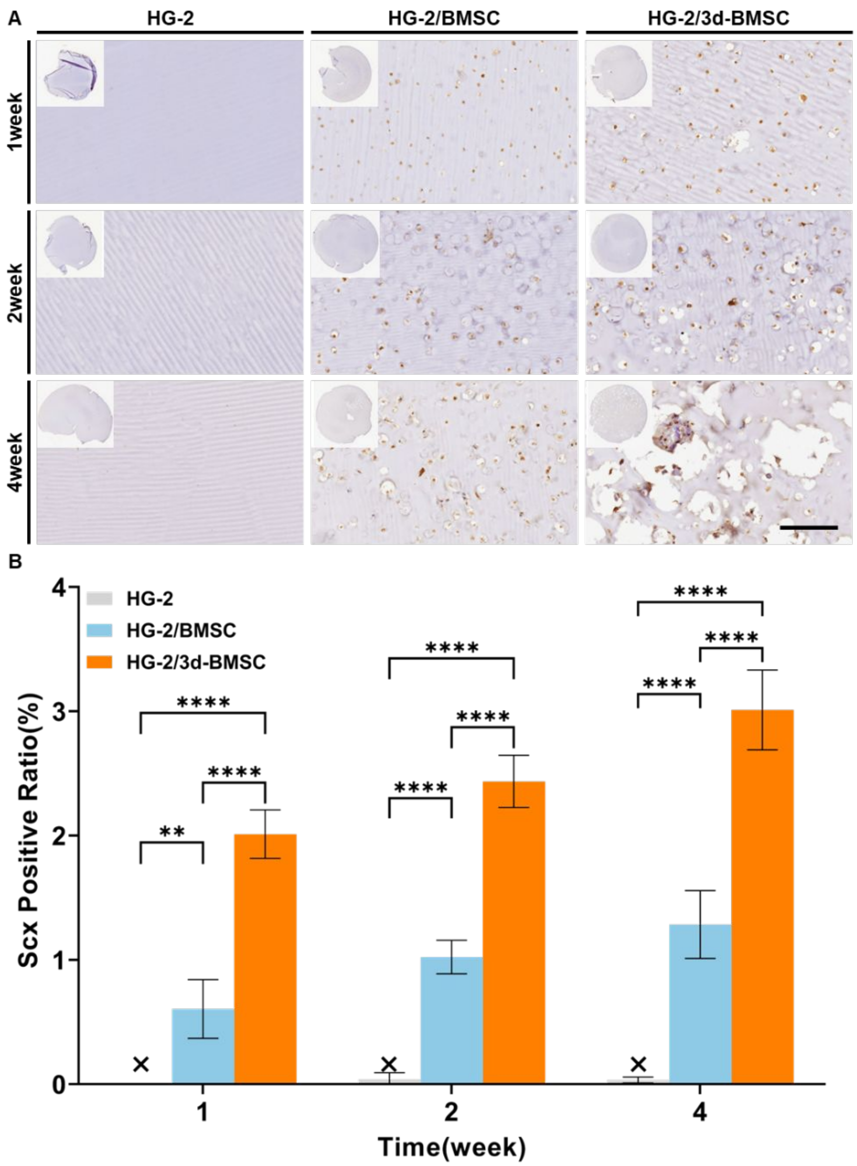

Figure S23. Typical Immunohistochemical staining images A) and quantitative analysis results B) of Scx proteins of different groups at 1, 2, and 4 weeks in vivo, one-way analysis of variance, one-tailed**.** Scale bar = 200 μm. All data are expressed as mean ± SD (n=3, ***p* < 0.01, *****p* < 0.0001, ns means no significance). HG-2 group, ECM mimic hydrogel without BMSCs; HG-2/BMSC group, ECM mimic hydrogel with non-adherent BMSCs; HG-2/3d-BMSC group, ECM mimic hydrogel with adherent BMSCs.

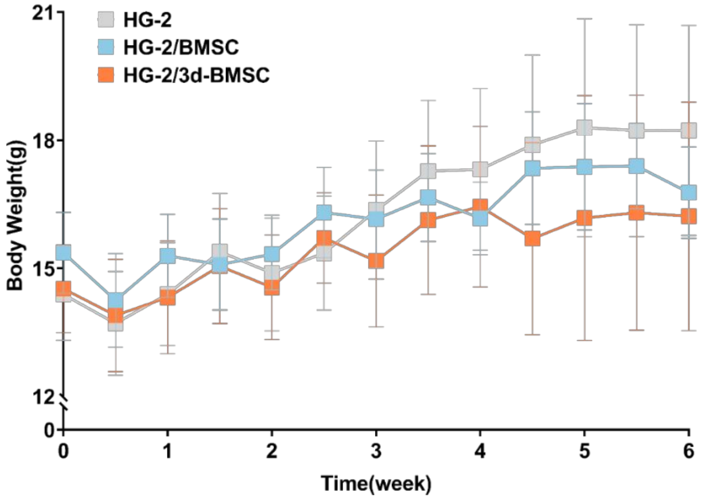

Figure S24. Body weight changes of mice implanted with different groups samples within 6 weeks. All data are represented as mean±SD (n=3). HG-2 group samples, ECM mimic hydrogel without BMSCs; HG-2/BMSC group samples, ECM mimic hydrogel with non-adherent BMSCs; HG-2/3d-BMSC group samples, ECM mimic hydrogel with adherent BMSCs.

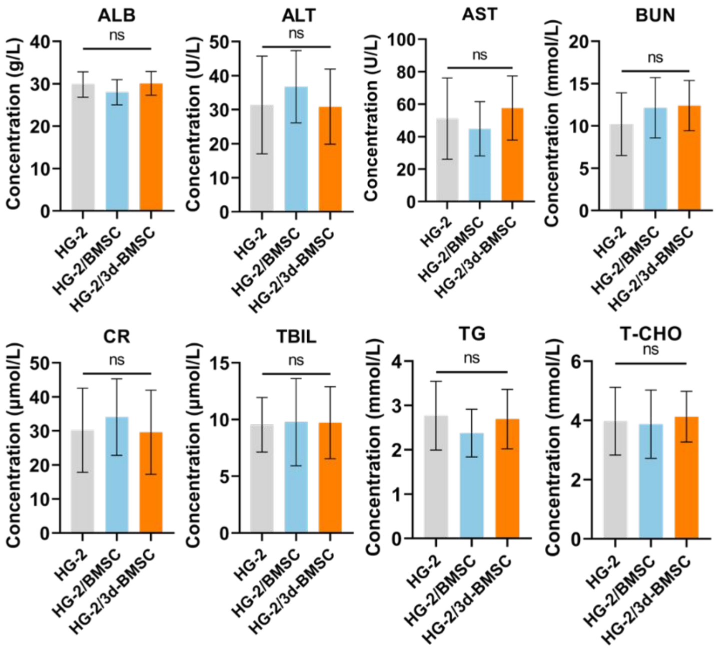

Figure S25. Biochemical parameters in the serum of mice implanted with different groups samples, one-way analysis of variance, two-tailed**.** All data are represented as mean±SD (n=3). ALB, albumin. ALT, alanine aminotransferase. AST, aspartate aminotransferase. BUN, blood urea nitrogen. CR, creatinine. TBIL, total bilirubin. TG, triglyceride. T-CHO, total cholesterol. HG-2 group samples, ECM mimic hydrogel without BMSCs; HG-2/BMSC group samples, ECM mimic hydrogel with non-adherent BMSCs; HG-2/3d-BMSC group samples, ECM mimic hydrogel with adherent BMSCs.

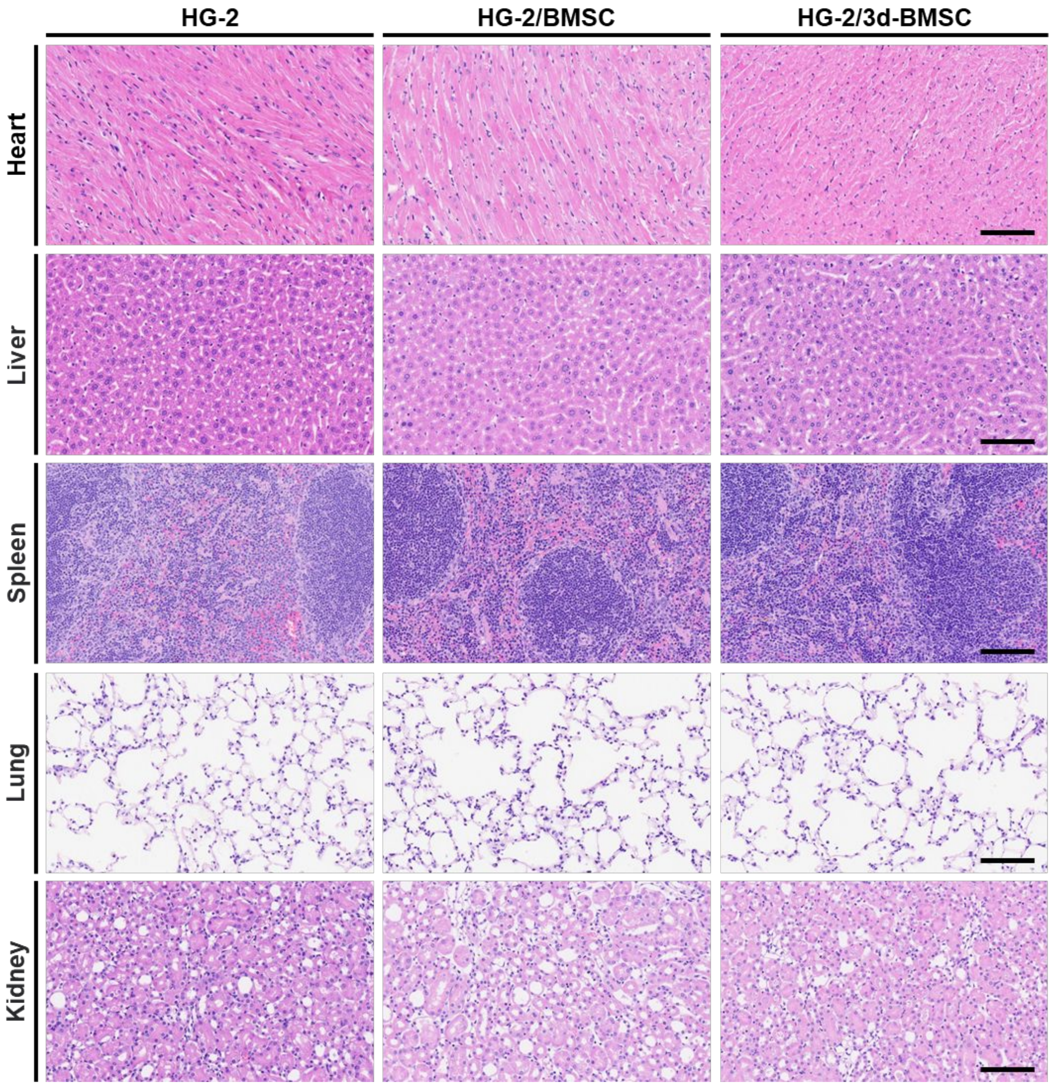

Figure S26. H&E staining of the hearts, livers, spleens, lungs, and kidneys of mice implanted with different groups samples. Scale bar = 100 μm. HG-2 group samples, ECM mimic hydrogel without BMSCs; HG-2/BMSC group samples, ECM mimic hydrogel with non-adherent BMSCs; HG-2/3d-BMSC group samples, ECM mimic hydrogel with adherent BMSCs.

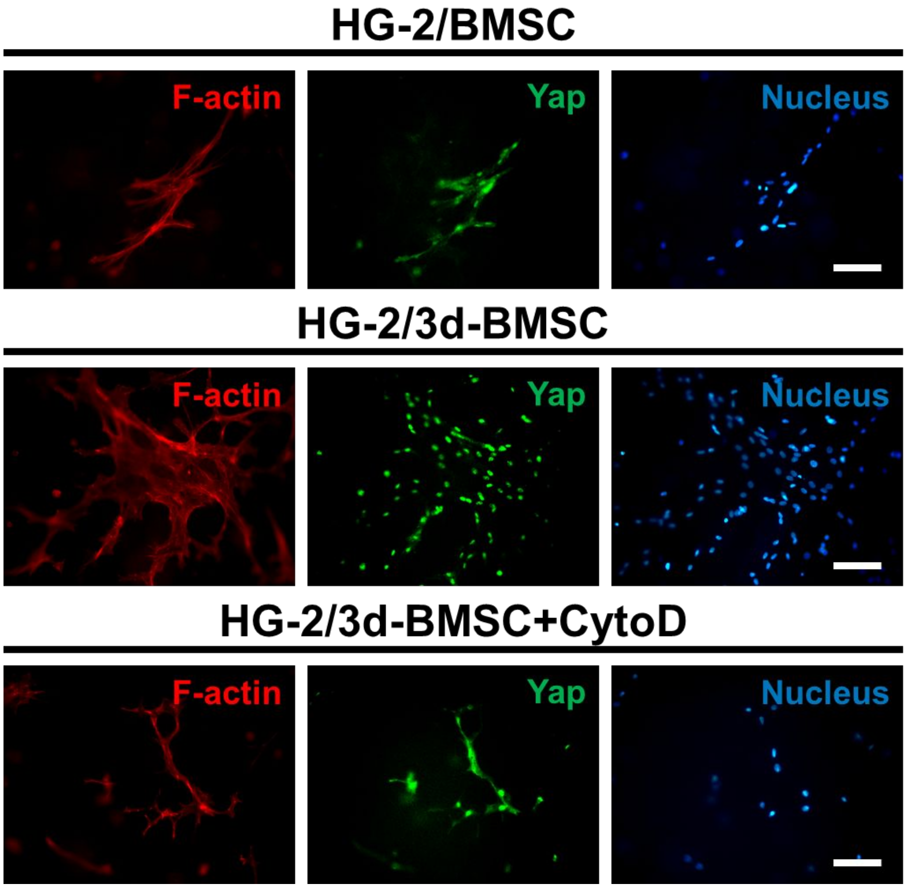

Figure S27. Immunofluorescence staining of Yap protein in different groups at Day3 after osteogenic differentiation induction. Scale bar=100 μm. HG-2/BMSC group, ECM mimic hydrogel with non-adherent BMSCs; HG-2/3d-BMSC group, ECM mimic hydrogel with adherent BMSCs; HG-2/3d-BMSC+CytoD group, ECM mimic hydrogel with adherent BMSCs and treated with 0.2 μM CytoD.

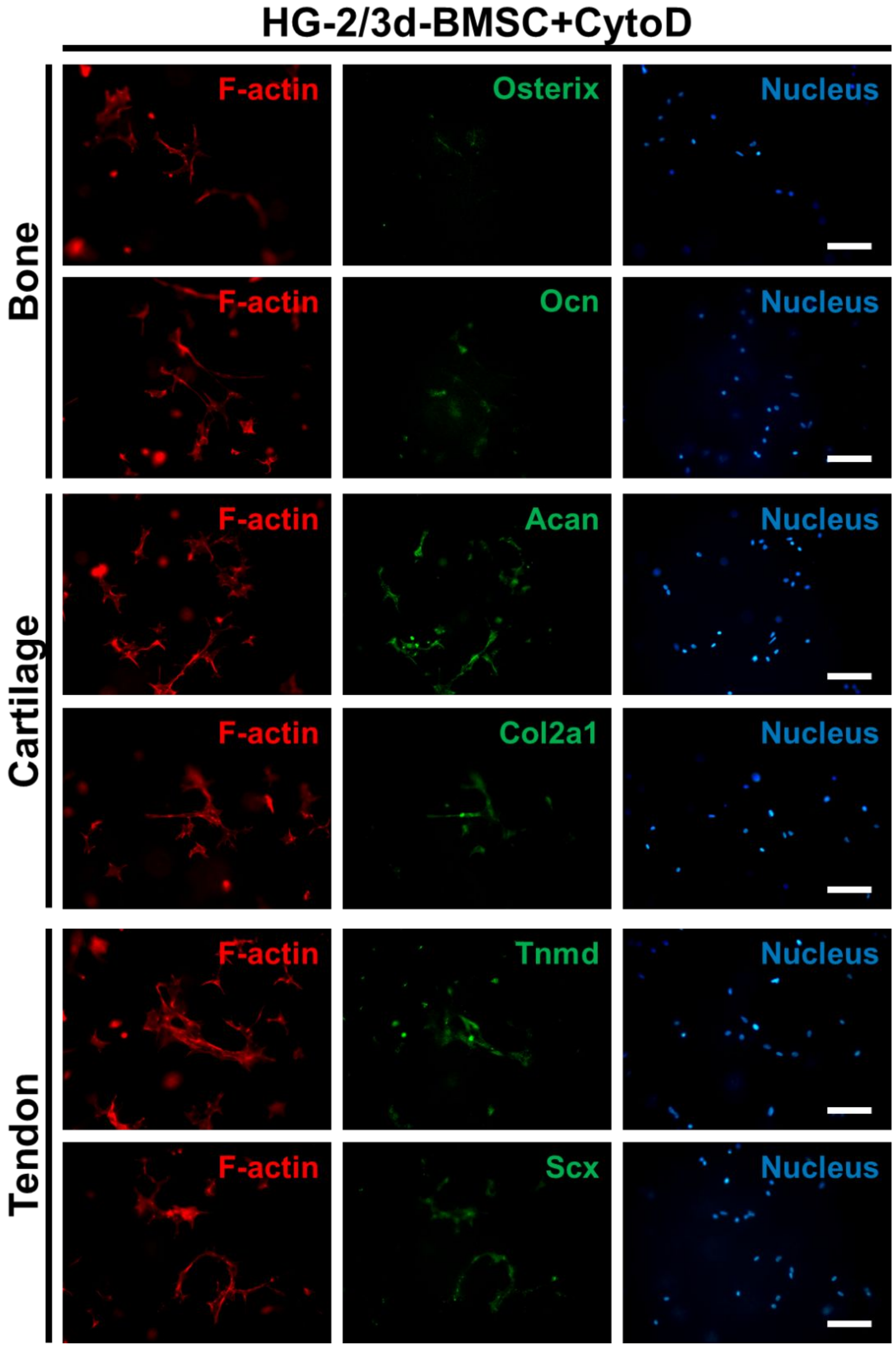

Figure S28. Immunofluorescence images of typical bone regeneration-related proteins (Ocn, Osterix), cartilage regeneration-related proteins (Col2a1, Acan) and tendon regeneration-related proteins (Scx, Tnmd) expressions in HG-2/3d-BMSC+CytoD group at Day3 after osteogenic differentiation induction. Scale bar=100 μm. HG-2/3d-BMSC+CytoD group, ECM mimic hydrogel with adherent BMSCs and treated with 0.2 μM CytoD.

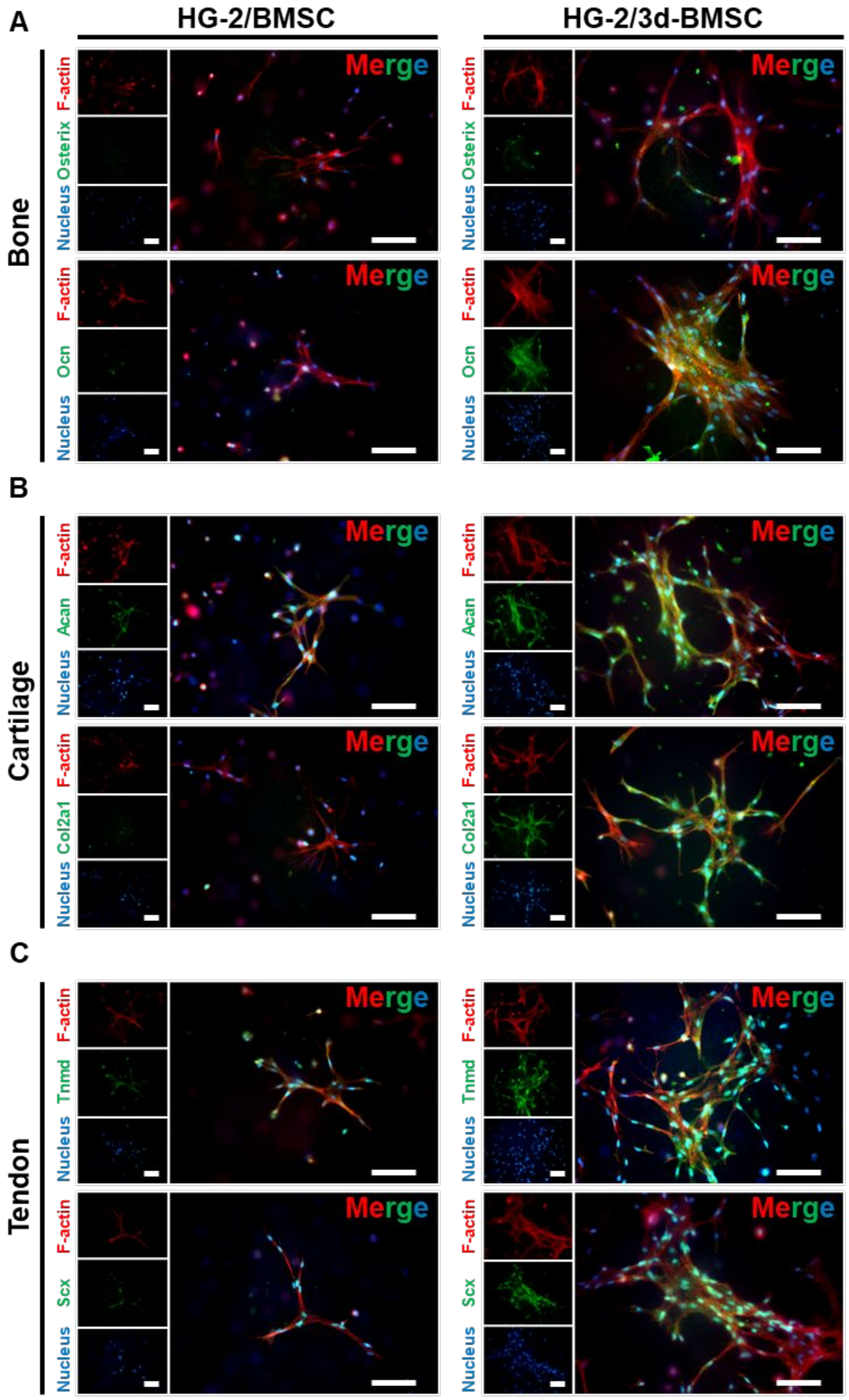

Figure S29. Multidirectional differentiation abilities of BMSCs in different groups after osteogenic differentiation induction for 3 days. A) Immunofluorescence images of typical bone regeneration-related proteins. B) Immunofluorescence images of typical cartilage regeneration-related proteins. C) Immunofluorescence images of tendon regeneration-related proteins. Scale bar = 100 μm. HG-2/BMSC group, ECM mimic hydrogel with non-adherent BMSCs; HG-2/3d-BMSC group, ECM mimic hydrogel with adherent BMSCs.

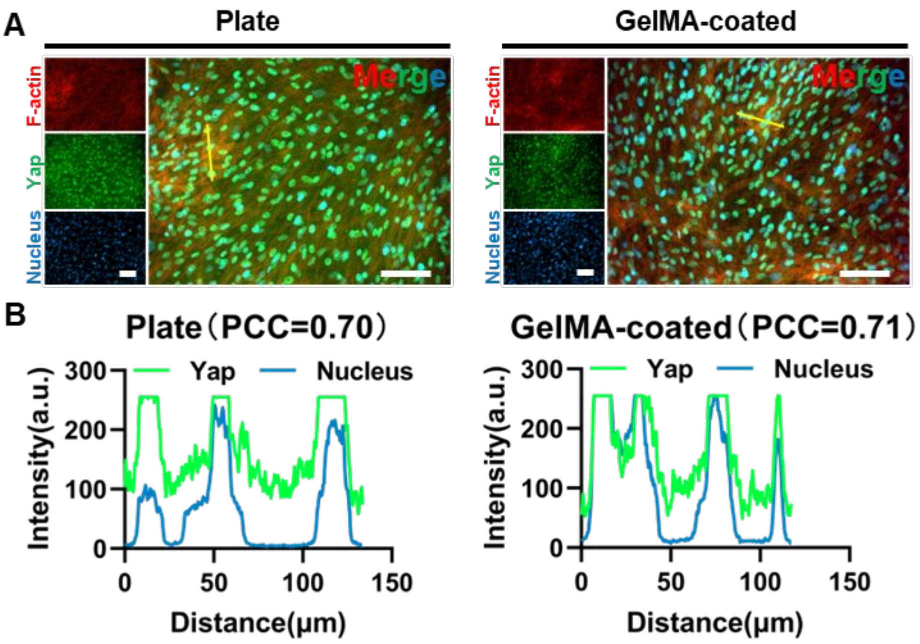

Figure S30. Cell adhesion-based spatial mechanical stimulation from ECM mimic hydrogel rather than ECM mimic hydrogel itself can increase Yap nuclear-cytoplasmic translocation. A, B) Immunofluorescence staining and co-localization analysis of Yap protein and nucleus of BMSCs in different groups at Day 3 after osteogenic differentiation induction, the yellow line area is analyzed. Scale bar = 100 μm. Plate group, BMSCs cultured on the plate; GelMA-coated group, BMSCs cultured on the ECM mimic hydrogel. PCC, Pearson's Correlation Coefficient.

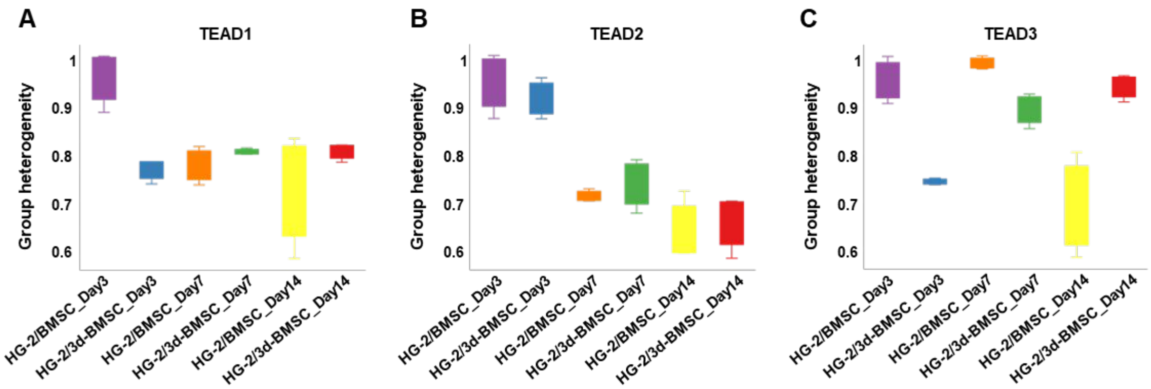

Figure S31. RNA-seq analysis showed the expression level of A) TEAD1, B) TEAD2 and C) TEAD3 gene of BMSCs in different groups after osteogenic differentiation induction for different times. HG-2/BMSC group, ECM mimic hydrogel with non-adherent BMSCs; HG-2/3d-BMSC group, ECM mimic hydrogel with adherent BMSCs.

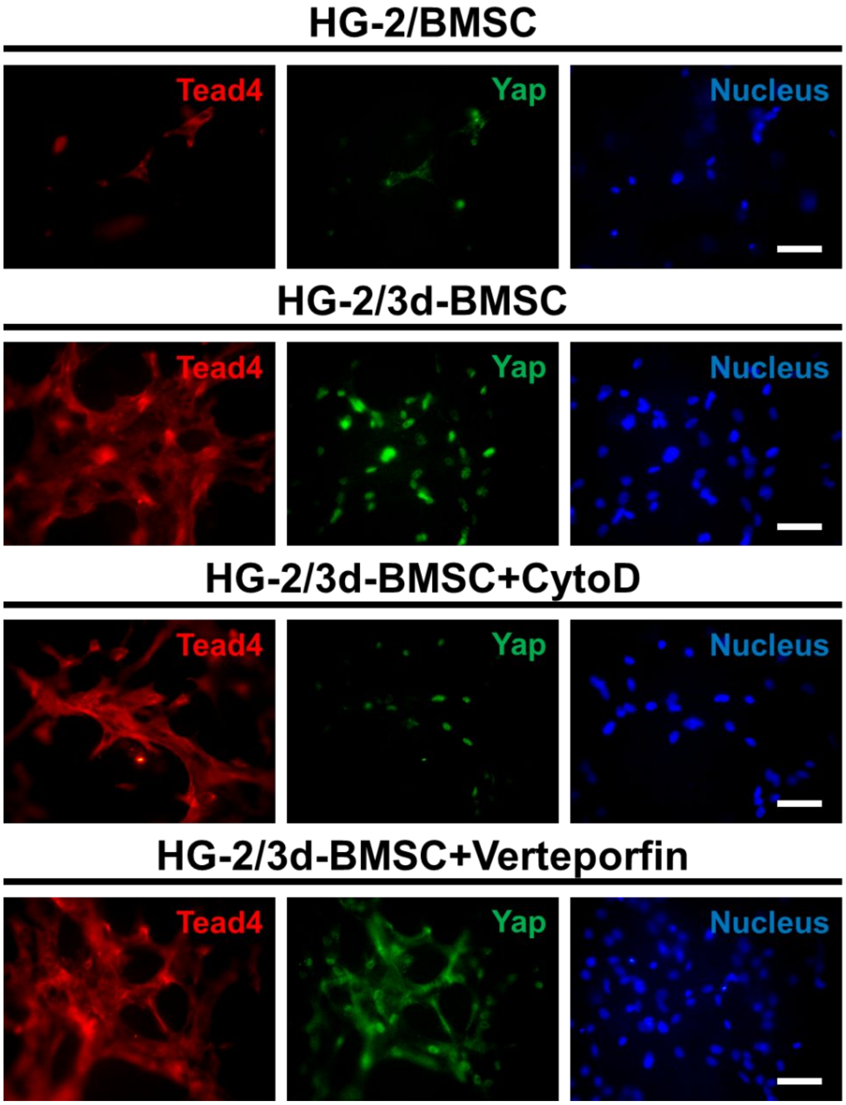

Figure S32. Immunofluorescence staining of Tead4 protein, Yap protein and nucleus of BMSCs in different groups at Day3 after osteogenic differentiation induction, Scale bar = 50 μm. HG-2/BMSC group, ECM mimic hydrogel with non-adherent BMSCs; HG-2/3d-BMSC group, ECM mimic hydrogel with adherent BMSCs; HG-2/3d-BMSC+CytoD group, ECM mimic hydrogel with adherent BMSCs and treated with 0.2 μM CytoD; HG-2/3d-BMSC+Verteporfin group, ECM mimic hydrogel with adherent BMSCs and treated with 2 μM Verteporfin.

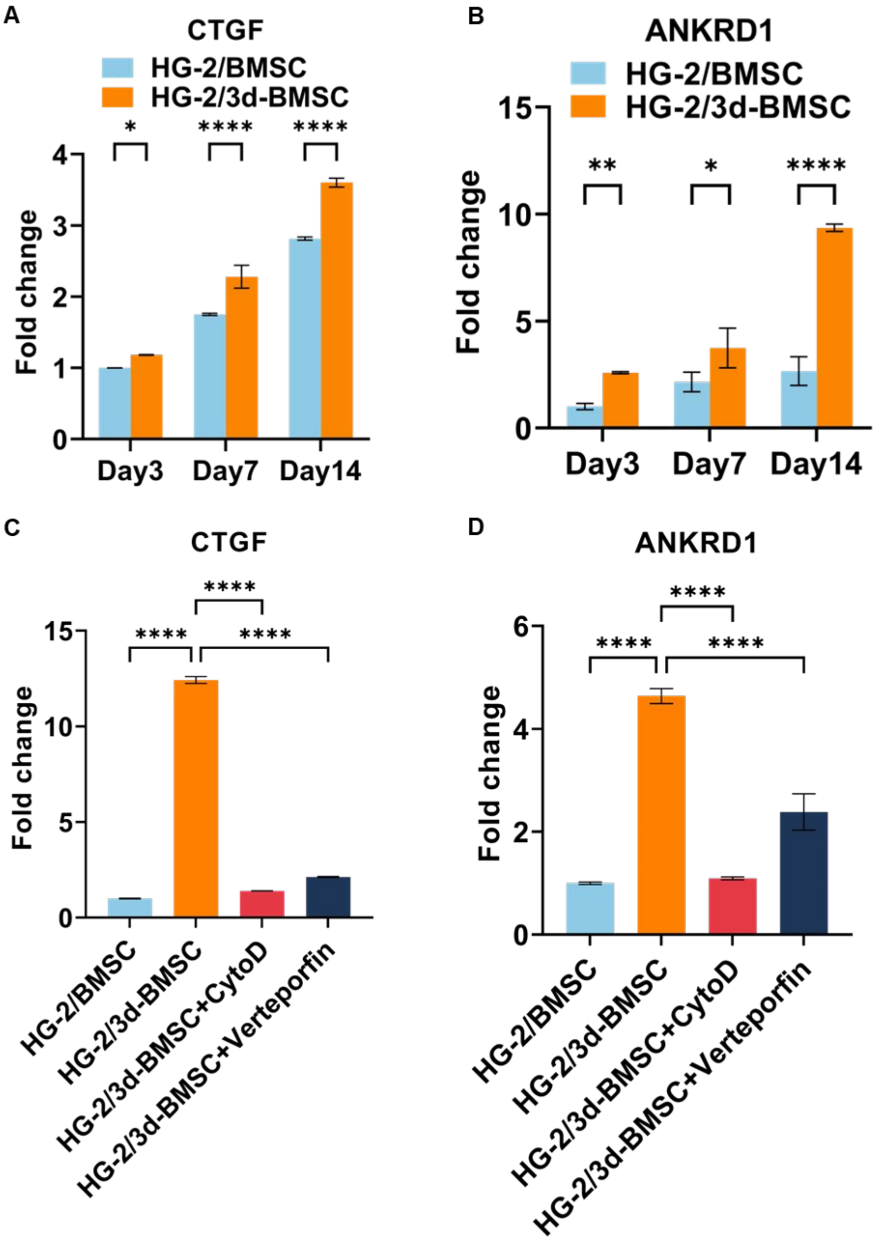

Figure S33. The expression of YAP downstream target genes. A, B) The expression of CTGF and ANKRD1 gene in BMSCs in different groups at various times after osteogenic differentiation induction, independent sample T test, one-tailed. C, D) qRT-PCR result showed expression of CTGF and ANKRD1 gene in BMSCs with different inhibitors at Day3 after osteogenic differentiation induction, one-way analysis of variance, one-tailed. All data are presented as mean±SD (n=3, *p <0.05, **p <0.01, ****p < 0.0001). HG-2/BMSC group, ECM mimic hydrogel with non-adherent BMSCs; HG-2/3d-BMSC group, ECM mimic hydrogel with adherent BMSCs; HG-2/3d-BMSC+CytoD group, ECM mimic hydrogel with adherent BMSCs and treated with 0.2 μM CytoD; HG-2/3d-BMSC+Verteporfin group, ECM mimic hydrogel with adherent BMSCs and treated with 2 μM Verteporfin.

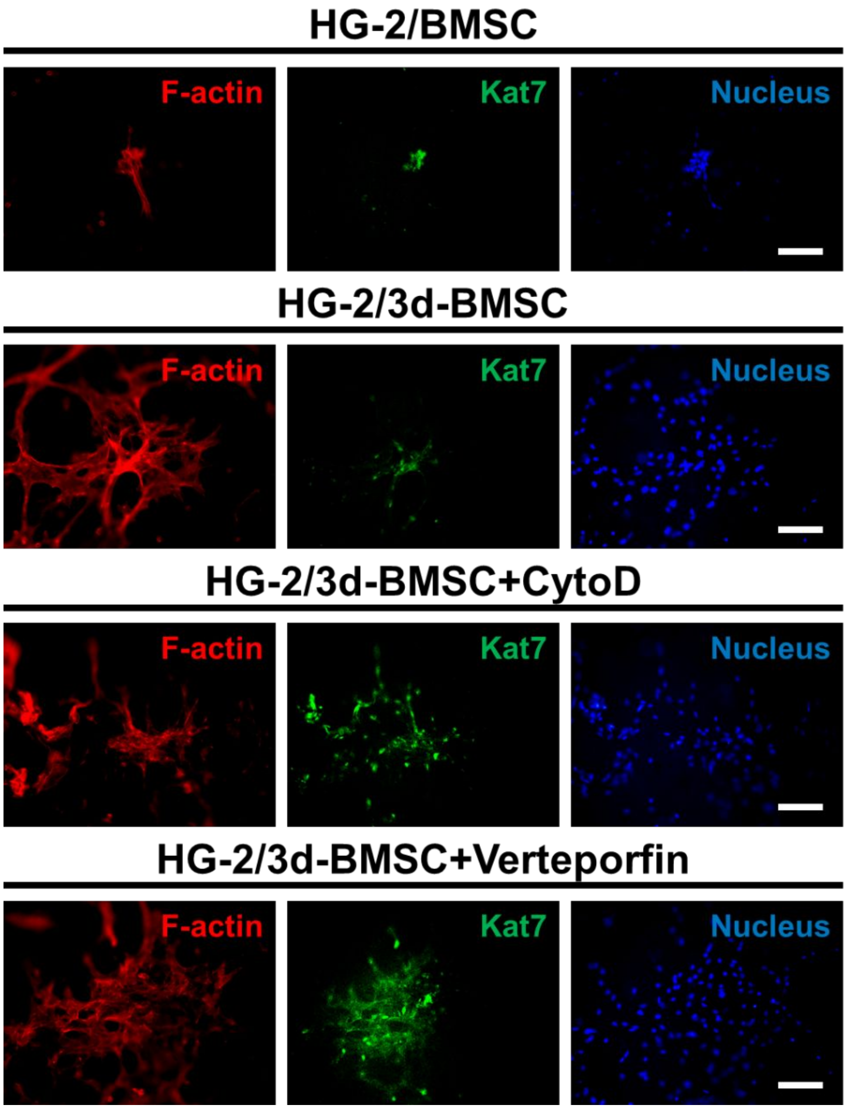

Figure S34. Immunofluorescence staining of Kat7 protein expression of BMSCs in different groups at Day3 after osteogenic differentiation induction, Scale bar = 100 μm. HG-2/BMSC group, ECM mimic hydrogel with non-adherent BMSCs; HG-2/3d-BMSC group, ECM mimic hydrogel with adherent BMSCs; HG-2/3d-BMSC+CytoD group, ECM mimic hydrogel with adherent BMSCs and treated with 0.2 μM CytoD; HG-2/3d-BMSC+Verteporfin group, ECM mimic hydrogel with adherent BMSCs and treated with 2 μM Verteporfin.

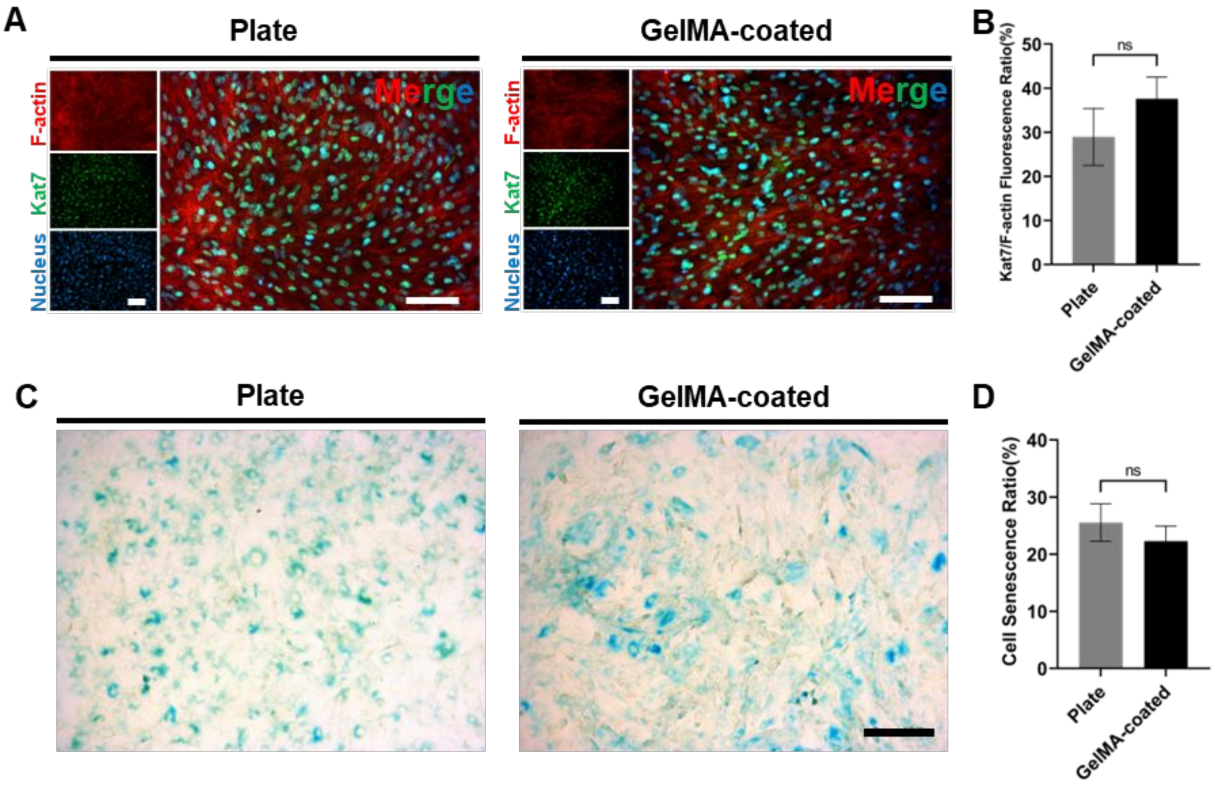

Figure S35. Cell adhesion-based spatial mechanical stimulation from ECM mimic hydrogel rather than ECM mimic hydrogel itself delays cell aging by suppressing Kat7 expression. A, B) Immunofluorescence staining and semi-quantitative analysis of Kat7 protein expression of BMSCs in different groups at Day 3 after osteogenic differentiation induction, independent sample T test, two-tailed. Scale bar = 100 μm. C, D) Assessment of cell senescence ratios in different groups at Day 3 after osteogenic differentiation induction with β-galactosidase staining, independent sample T test, two-tailed. Scale bar = 200 μm. All data are presented as mean±SD (n = 3, ns means no significance). Plate group, BMSCs cultured on the plate; GelMA-coated group, BMSCs cultured on the ECM mimic hydrogel.

Table S1. The sequence of primers used in this work.

Name	Sequence（5’-3’）
β-actin-Forward	GTAAAGACCTCTATGCCAACA
β-actin-Reverse	GGACTCATCGTACTCCTGCT
OSTERIX-Forward	TGACTGCCTGCCTAGTGTCTACA
OSTERIX-Reverse	TGGATGCCCGCCTTGT
OCN-Forward	GCCCTGACTGCATTCTGCCTCT
OCN-Reverse	TCACCACCTTACTGCCCTCCTG
ACAN-Forward	CCGCTGGTCTGATGGACACT
ACAN-Reverse	AGGTGTTGGGGTCTGTGCAA
COL2A1-Forward	CTGGTCCTTCCGGCCCTAGA
COL2A1-Reverse	GGATCGGGGCCCTTCTCTCT
SCX-Forward	AACACGGCCTTCACTGCGCTG
SCX-Reverse	CAGTAGCACGTTGCCCAGGTG
TNMD-Forward	CCAGACAAGCAAGCGAGGA
TNMD-Reverse	AACTTCCTATTAGACTCTCC
TEAD4-Forward	TGTCAGACGAAGGCAAGATGTATGG
TEAD4-Reverse	ACCTGGATGTGGCTGGAGACC
CTGF-Forward	CACACCGCACAGAACCACCAC
CTGF -Reverse	TGTAATGGCAGGCACAGGTCTTG
ANKRD1-Forward	CTGGGCATGTCGTGGAGGAAAC
ANKRD1-Reverse	GCAGCTTGTCTCGGGCACTG
KAT7-Forward	GCACTGAGGAACCCGCCTAT
KAT7-Reverse	ACCGCCTGTTCCGTTTCAGA

Table S2. The details of primary antibodies for immunofluorescence staining.

Name	Item Number	Host	Dilution Ratio
Anti-Osterix	ab209484	Rabbit	1:500
Anti-Ocn	23418-1-AP	Rabbit	1:500
Anti-Acan	13880-1-AP	Rabbit	1:500
Anti-Col2a1	28459-1-AP	Rabbit	1:500
Anti-Tnmd	ab203676	Rabbit	1:500
Anti-Scx	PA5-115874	Rabbit	1:500
Anti-Kat7	ab70183	Rabbit	1:500
anti-Yap	14074S	Rabbit	1:500
anti-Tead4	ab58310	Mouse	1:500
Actin-Tracker Red-555	C2203S	Rabbit	1:100
